# Supplementary material for: Enhanced carbon dioxide electrolysis at redox manipulated interfaces
Source: Nat Commun. 2019 Apr 4;10:1550. doi: 10.1038/s41467-019-09568-1 (PMC6449360; doi:10.1038/s41467-019-09568-1)
Supplement: Supplementary file 1 — Supplementary Information [file 41467_2019_9568_MOESM1_ESM.pdf]

Supplementary Information

Enhanced carbon dioxide electrolysis at redox manipulated interfaces

Wang et al.

Supplementary Figures

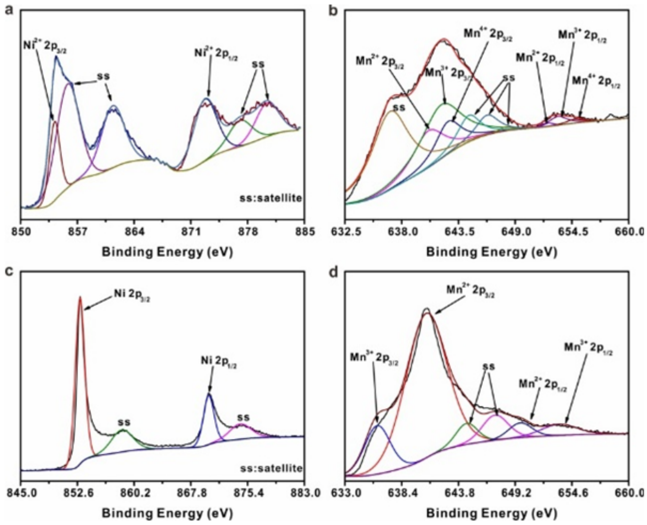

Supplementary Fig. 1. X-ray photoelectron spectroscopy (XPS) for Ni-MnO_x samples. Oxidized NiO/15%$NiMn_2O_4$ with (a) Ni and (b) Mn; XPS for reduced Ni/11%MnOx with (c) Ni and (d) Mn.

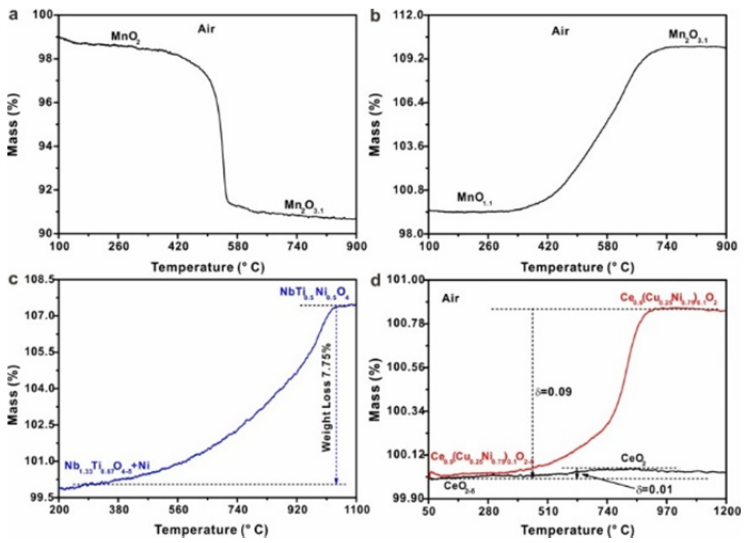

Supplementary Fig. 2. The thermogravimetric analysis of different samples. (a) MnO_2 powder heated in air to get end composition; (b) MnO_x obtained from $MnCO_3$ in pure H_2 at 800 °C and then heated in air to get end composition; (c) reduced $NbTi_{0.5}Ni_{0.5}O_4$ heated in air; (d) reduced CeO_2 and $Ce_{0.9}(Cu_{0.25}Ni_{0.75})_{0.1}O_{2-\delta}$ heated in air.

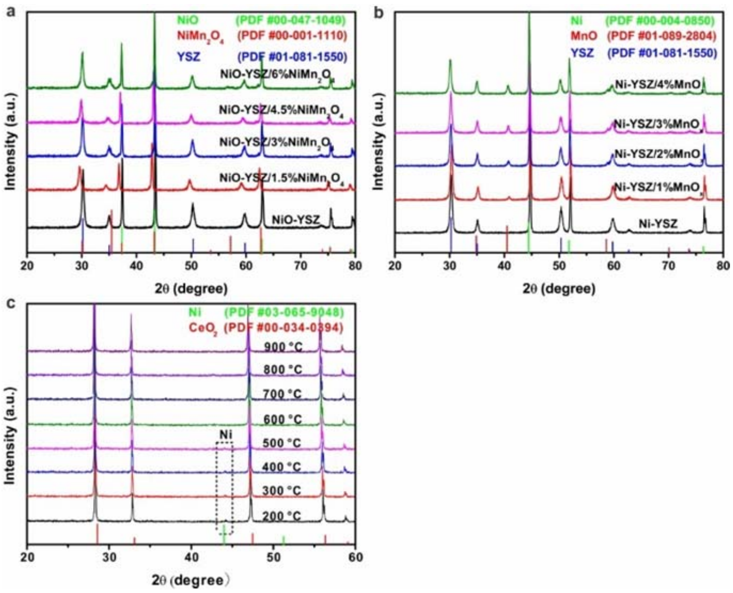

Supplementary Fig. 3. XRD of powder samples. (a) XRD of NiO-YSZ/x%$NiMn_2O_4$ (x=0-6); (b) XRD of reduced Ni-YSZ/y%MnO_x (y= 0-4); (c) *In situ* XRD of reduced $Ce_{0.9}Ni_{0.1}O_{2-\delta}$ tested during heating in air.

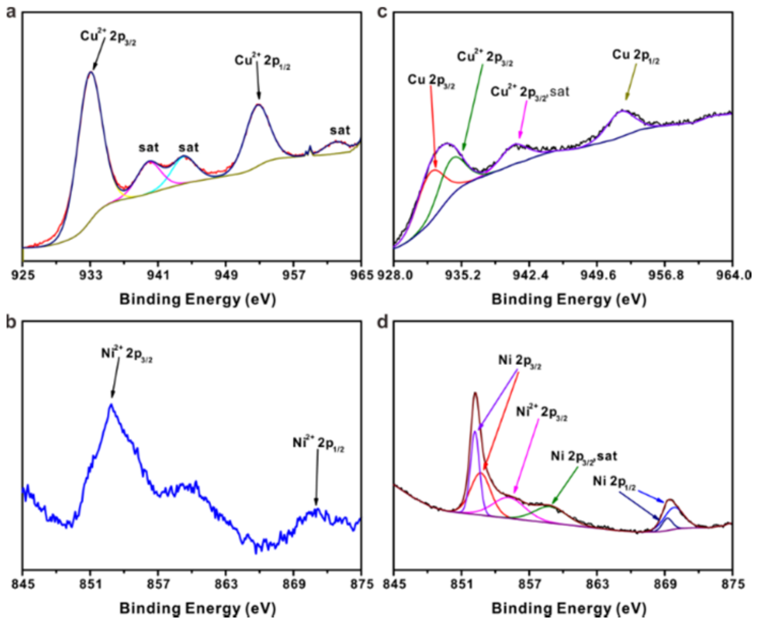

Supplementary Fig. 4. X-ray photoelectron spectroscopy (XPS) for $Ni_{0.75}Cu_{0.25}$-$CeO_{2-\delta}$ samples. XPS of $Ce_{0.9}(Cu_{0.25}Ni_{0.75})_{0.1}O_{2-\delta}$ with (a) Cu and (b) Ni; XPS of reduced $Ce_{0.9}(Cu_{0.25}Ni_{0.75})_{0.1}O_{2-\delta}$ with (c) Cu and (d) Ni.

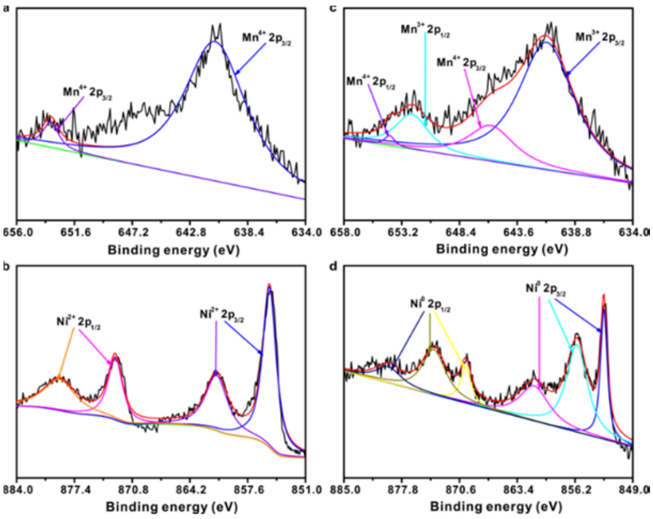

Supplementary Fig. 5. X-ray photoelectron spectroscopy (XPS) for Ni-$NbTi_{0.4}Mn_{0.1}O_4$ samples. XPS of $NbTi_{0.4}Mn_{0.1}Ni_{0.5}O_4$ with (a) Mn and (b) Ni; XPS of reduced $NbTi_{0.4}Mn_{0.1}Ni_{0.5}O_4$ with (c) Mn and (d) Ni.

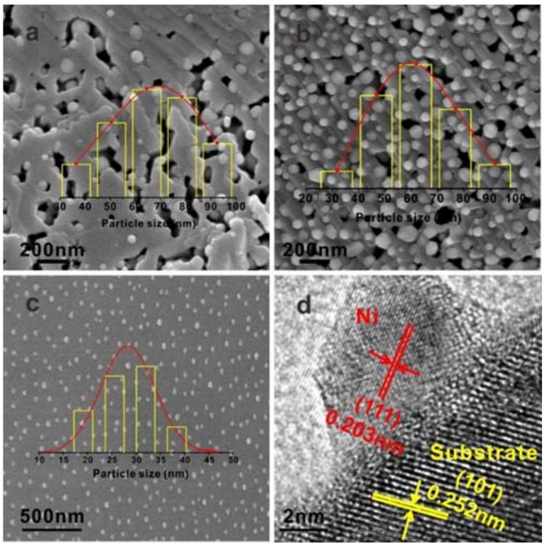

Supplementary Fig. 6. SEM of Ni-MnO_x and Ni-$NbTi_{0.4}Cr_{0.1}Ni_{0.5}O_4$ samples. Microstructure of (a) Ni/8%MnO_x, (b) Ni/15%MnO_x samples with particle size distribution. SEM (c) and TEM (d) of reduced $NbTi_{0.4}Cr_{0.1}Ni_{0.5}O_4$ with particle size distribution.

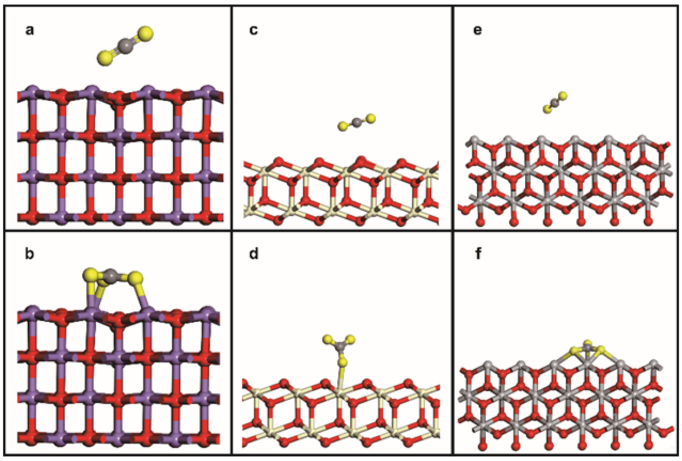

Supplementary Fig. 7. Structures of CO_2 species adsorbed on (a) MnO, (c) CeO_2 and (e) TiO_2 surfaces as well as structures of CO_3^{2-} species adsorbed on (b) MnO, (d) CeO_2 and (f) TiO_2 surfaces. Cerium in apricot cream, titanium in silvery white, manganese in purple, carbon in grey, oxygen in red and oxygen of CO_2 species and CO_3^{2-} species in yellow for clear.

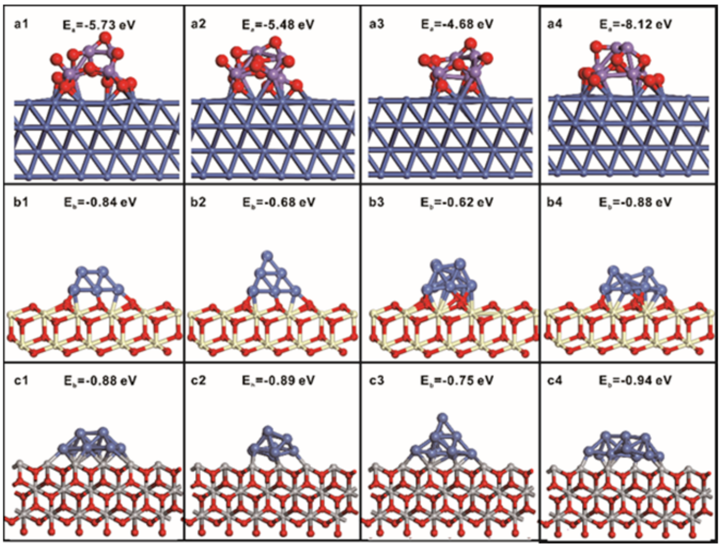

Supplementary Fig. 8. Adsorption energies of MnO_x on Ni(111) surface and binding energies of different clusters on CeO_2(111) surface and TiO_2(101) surface. (a1-a2) different Mn_6O_{12} shapes while (a3-a4) different Mn_6O_9 shapes on the Ni(111) surface. (b1-b4) different Ni clusters shapes on the CeO_2(111) surface. (c1-c4) different Ni clusters shapes on the TiO_2(101) surface. Nickel in blue, cerium in apricot cream, titanium in silvery white, manganese in purple and oxygen in red.

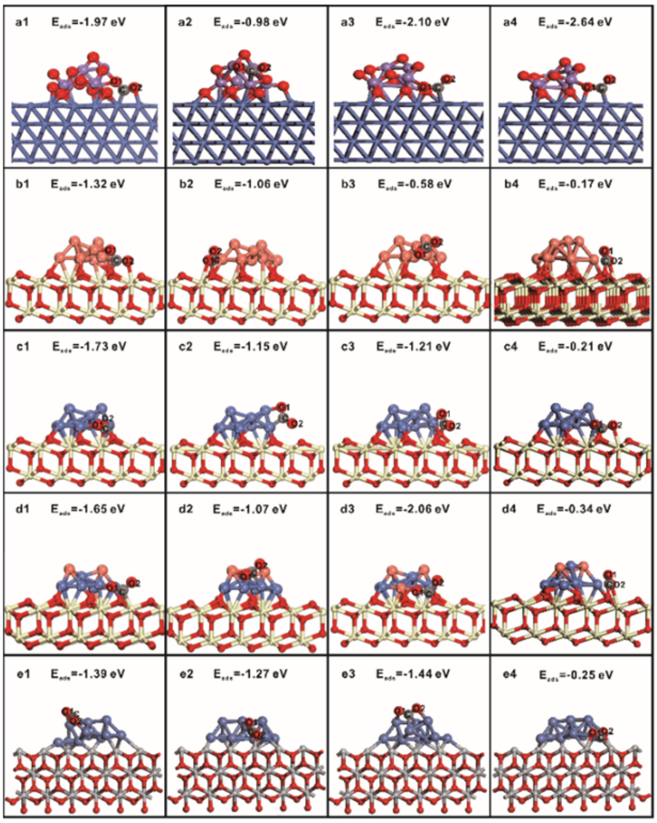

Supplementary Fig. 9. The adsorption configurations of CO_2 on MnO_x/Ni(111), M/CeO_2(111) and Ni/TiO_2(101) systems, respectively. (a1-a2) Mn_6O_{12}/Ni(111), (a3) Mn_6O_{10}/Ni(111) and (a4) Mn_6O_9/Ni(111) systems; (b-d) M/CeO_2(111) systems, M represents Cu, Ni and Ni-Cu clusters respectively; (e1-e4) Ni/TiO_2(101) systems. Nickel in blue, copper in orange, cerium in apricot cream, titanium in silvery white, manganese in purple, carbon in grey and oxygen in red.

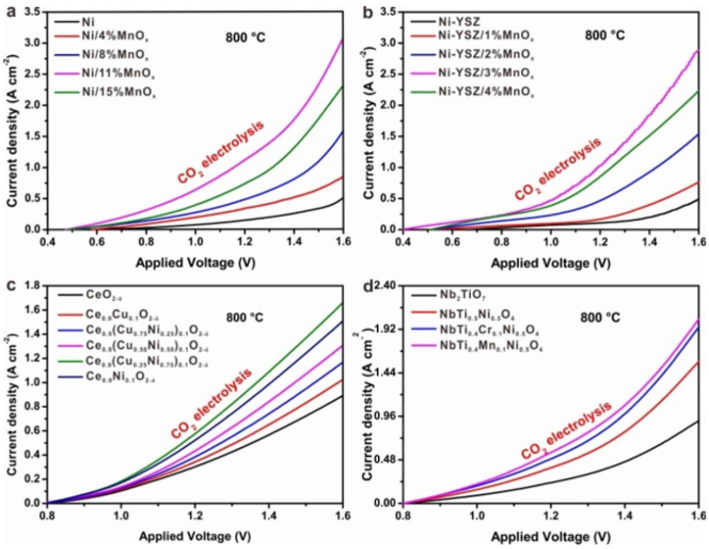

Supplementary Fig. 10. *I-V* curves of three system samples measured at 800 °C. (a) and (b) was tested in 80%CO_2/2%CO/18%Ar atmosphere. (c) and (d) was tested in CO_2 atmosphere.

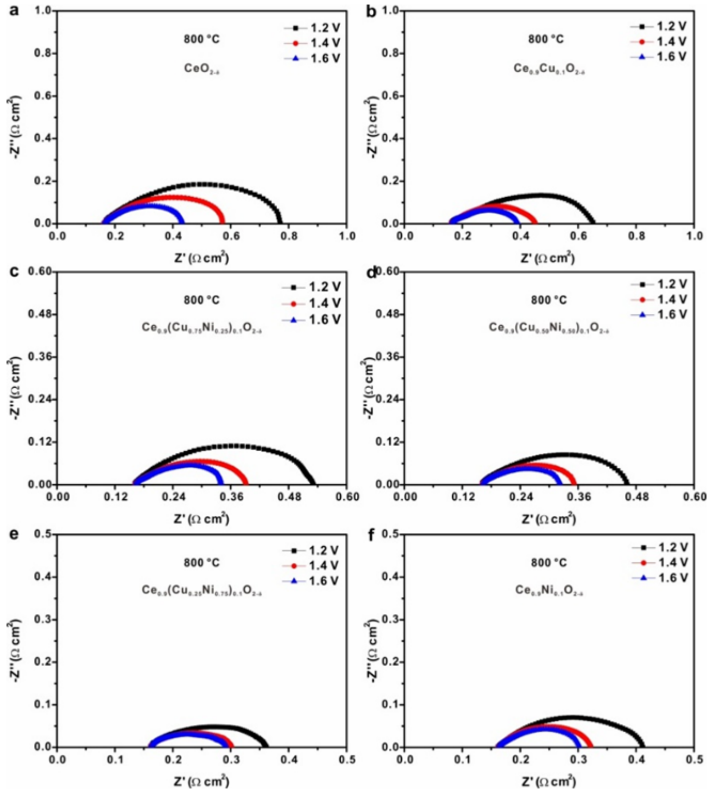

Supplementary Fig. 11. *In situ* AC impedance of single cells tested with different cathodes. (a) $CeO_{2-\delta}$, (b) $Ce_{0.9}Cu_{0.1}O_{2-\delta}$, (c) $Ce_{0.9}(Cu_{0.75}Ni_{0.25})_{0.1}O_{2-\delta}$, (d) $Ce_{0.9}(Cu_{0.50}Ni_{0.50})_{0.1}O_{2-\delta}$, (e) $Ce_{0.9}(Cu_{0.25}Ni_{0.75})_{0.1}O_{2-\delta}$ and (f) $Ce_{0.9}Ni_{0.1}O_{2-\delta}$ were tested at 800 °C. The test atmosphere was CO_2.

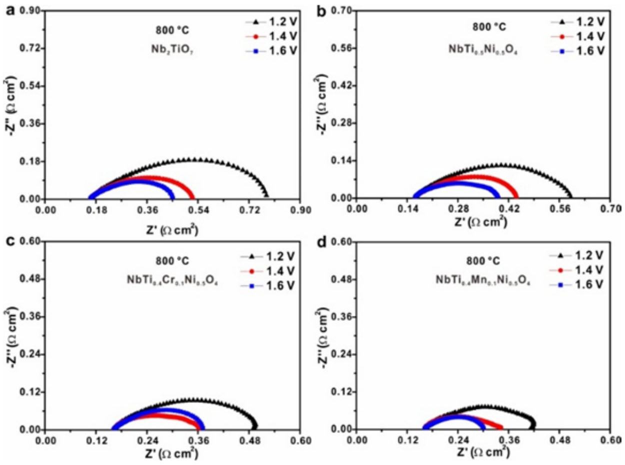

Supplementary Fig. 12. *In situ* AC impedance of single cells tested with different cathodes. (a) Ni_2TiO_7, (b) $NbTi_{0.5}Ni_{0.5}O_4$, (c) $NbTi_{0.4}Cr_{0.1}Ni_{0.5}O_4$, and (d) $NbTi_{0.4}Mn_{0.1}Ni_{0.5}O_4$ were tested at 800 °C. The test atmosphere was CO_2.

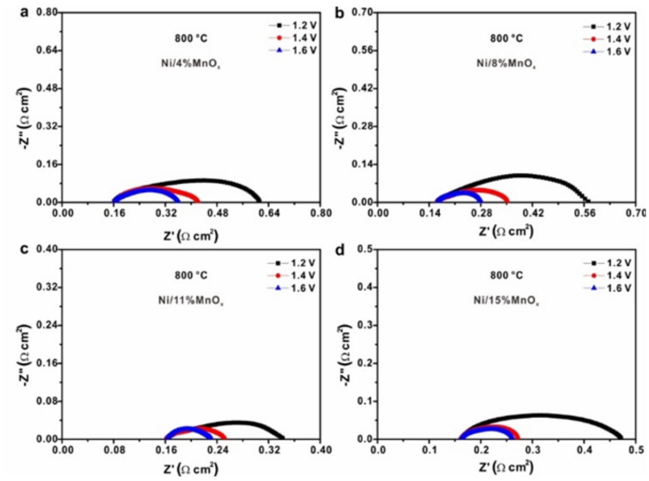

Supplementary Fig. 13. *In situ* AC impedance of single cells with different cathodes. (a) Ni/4%MnO_x, (b) Ni/8% MnO_x, (c) Ni/11%MnO_x, and (d) Ni/15%MnO_x tested at 800 °C. The test atmosphere was 80%CO_2/2%CO/18%Ar.

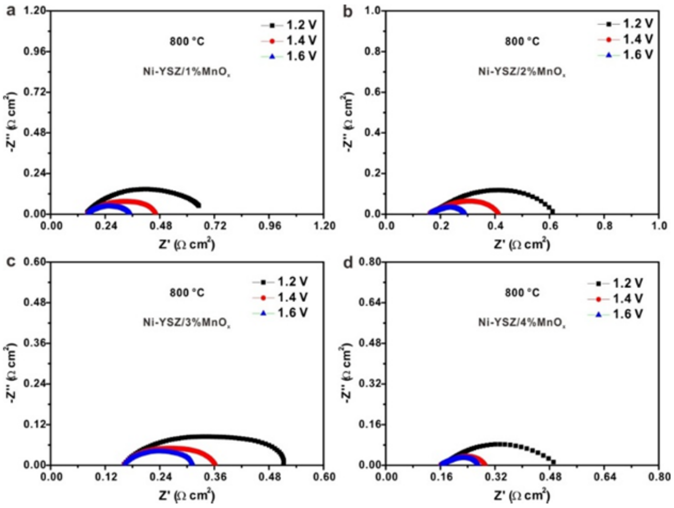

Supplementary Fig. 14. *In situ* AC impedance of single cells with different cathodes: (a) Ni-YSZ/1%MnO_x, (b) Ni-YSZ/2% MnO_x, (c) Ni-YSZ/3% MnO_x, and (d) Ni-YSZ/4% MnO_x. The test atmosphere was 80%CO_2/2%CO/18%Ar.

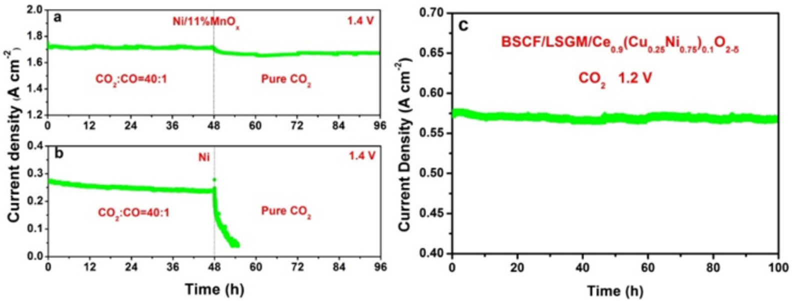

Supplementary Fig. 15. Long-term performance of single cells with different cathodes fed with CO_2 (50mL min^{-1}) at 800 °C: (a) Ni/11%MnO_x, (b) Ni and (c) $Ce_{0.9}(Cu_{0.25}Ni_{0.75})_{0.1}O_{2-\delta}$.

Supplementary Tables

Supplementary Table 1. Vibrational frequencies in cm^{-1} of CO_2 species and CO_3^{2-} species adsorbed on MnO, CeO_2 and TiO_2 surfaces.

system	species	parameter	Figure	C-O (Å)	υ (cm^{-1})
MnO	CO_2	experimental	-	-	2361
		computational	7a	1.17	2372
	CO_3^{2-}	experimental	-	-	1382
		computational	7b	1.31	1364
CeO_2	CO_2	experimental	-	-	2360
		computational	7c	1.17	2372
	CO_3^{2-}	experimental	-	-	1389
		computational	7d	1.26	1390
TiO_2	CO_2	experimental	-	-	2359
		computational	7e	1.18	2355
	CO_3^{2-}	experimental	-	-	1397
		computational	7f	1.29	1398

Supplementary Table 2. Geometrical parameters and calculated adsorption energies of CO_2 species on MnO_x/Ni(111) surface. Mn_6O_{12}/Ni-I and Mn_6O_{12}/Ni-II are the adsorption configurations of Figure 5 a1 and a4, Mn_6O_9/Ni-I and Mn_6O_9/Ni-II are the adsorption configurations of Figure 5 b1 and b4.

parameter	CO_2	Mn_6O_{12}/Ni-I	Mn_6O_{12}/Ni-II	Mn_6O_{10}/Ni	Mn_6O_9/Ni-I	Mn_6O_9/Ni-II
C-Ni (Å)	-	-	1.87	1.91	1.89	-
C-Mn (Å)	-	2.03	-	-	-	2.19
Mn-O1 (Å)	-	1.86	2.05	1.96	1.94	-
Ni-O1 (Å)	-	-	-	2.00	2.00	-
Ni-O2 (Å)	-	-	2.03	-	-	-
C-O1 (Å)	1.18	1.33	1.27	1.38	1.39	1.38
C-O2 (Å)	1.18	1.21	1.28	1.21	1.21	1.21
O-C-O (°)	180	130.4	127.2	124.4	124.0	124.0
E_{ads} (eV)	-	-0.98	-1.97	-2.10	-2.64	-1.62

Supplementary Table 3. Geometrical parameters and calculated adsorption energies of CO_2 species on the M/CeO_2(111) systems. (M is expressed as Cu, Ni or Ni-Cu clusters).

parameter	CO_2	Cu/CeO_2	Ni/CeO_2	Ni-Cu/CeO_2	Cu/CeO_{2-x}	Ni/CeO_{2-x}	(Ni-Cu)/CeO_{2-x}
C-O (Å)	-	1.36	1.36	1.36	-	-	-
C-Ni (Å)	-	-	-	-	-	1.85	1.85
C-Cu (Å)	-	1.93	-	-	1.94	-	-
Ni-O1 (Å)	-	-	-	-	-	-	1.86
Ni-O2 (Å)	-	-	1.86	-	-	-	-
Cu-O1 (Å)	-	1.84	-	1.84	-	-	-
Ce-O1 (Å)	-	-	-	-	-	-	2.57
Ce-O2 (Å)	-	2.41	2.43	-	2.50/2.55	2.58/2.60	2.59/2.62
C-O1 (Å)	1.18	1.28	1.27	1.27	1.24	1.23	1.23
C-O2 (Å)	1.18	1.27	1.28	1.21	1.36	1.35	1.35
O-C-O (°)	180	123.3	123.2	124.0	117.5	116.3	123.2
E_{ads} (eV)	-	-1.32	-1.73	-2.06	-1.83	-1.90	-2.18

Supplementary Table 4. Geometrical parameters and calculated adsorption energies of CO_2 species on Ni/TiO_2(101) systems. TiO_2-I to TiO_2-IV are the adsorption configurations of Figure 9 e1 to e4, TiO_{2-x}-I and TiO_{2-x}-II are the adsorption configurations of Figure 5 f1 and f4.

parameter	CO_2	TiO_2-I	TiO_2-II	TiO_2-III	TiO_2-IV	TiO_{2-x}-I	TiO_{2-x}-II
C-Ni (Å)	-	1.93	2.06	1.96	-	1.87	1.78/1.87
O1-Ni (Å)	-	1.97	1.97	1.95	-	2.01	-
O2-Ni (Å)	-	1.97	-	1.95	-	1.99	-
O-Ti (Å)	-	-	2.15	-	2.04/1.99	-	1.67
C-O1 (Å)	1.18	1.28	1.28	1.29	1.20	1.26	1.22
C-O2 (Å)	1.18	1.27	1.28	1.30	1.28	1.29	3.85
O-C-O (°)	180	130.8	126.0	127.2	134.3	131.2	106.8
E_{ads} (eV)	-	-1.39	-1.27	-1.44	-0.25	-1.16	-1.94

Supplementary Table 5. The energy values and adsorption energy of CO_2 with different cut-off energy. TiO_2-I to TiO_2-IV are the adsorption configurations of Figure 9 e1 to e4.

Cut-off (eV)	300	350	400	450	500	550	600	650
$\mathbf{E_{CO_2}}$ (eV)	-23.16	-23.02	-23.98	-22.95	-22.94	-22.95	-22.96	-22.97
$\mathbf{E_{TiO_2}}$ (eV)	-1309.35	-1300.10	-1296.86	-1296.48	-1296.18	-1296.19	-1296.47	-1296.77
$\mathbf{E_{TiO_2}}$-I (eV)	-1333.91	-1324.54	-1322.24	-1320.82	-1320.45	-1320.53	-1320.82	-1321.13
$\mathbf{E_{TiO_2}}$-II (eV)	-1333.79	-1324.02	-1322.12	-1320.70	-1320.19	-1320.40	-1320.63	-1321.01
$\mathbf{E_{TiO_2}}$-III (eV)	-1333.97	-1324.60	-1322.28	-1320.87	-1320.50	-1320.58	-1320.87	-1321.15
$\mathbf{E_{TiO_2}}$-IV (eV)	-1332.82	-1323.41	-1321.09	-1319.68	-1319.30	-1319.38	-1319.68	-1319.98
$\mathbf{E_{ads}}$-I (eV)	-1.41	-1.42	-1.40	-1.39	-1.33	-1.39	-1.39	-1.39
$\mathbf{E_{ads}}$-II (eV)	-1.29	-0.90	-1.29	-1.27	-1.07	-1.26	-1.20	-1.27
$\mathbf{E_{ads}}$-III (eV)	-1.47	-1.47	-1.45	-1.44	-1.37	-1.43	-1.44	-1.41
$\mathbf{E_{ads}}$-IV (eV)	-0.32	-0.29	-0.26	-0.25	-0.18	-0.24	-0.25	-0.24
