# Supplementary material for: Enhanced carbon dioxide electrolysis at redox manipulated interfaces
Source: Nat Commun. 2019 Apr 4;10:1550. doi: 10.1038/s41467-019-09568-1 (PMC6449360; doi:10.1038/s41467-019-09568-1)
Supplement: Supplementary file 3 — Source Data [file 41467_2019_9568_MOESM3_ESM.zip › Source Data-20190315/Figure 5/Figure 5.pptx]

## Slide 1
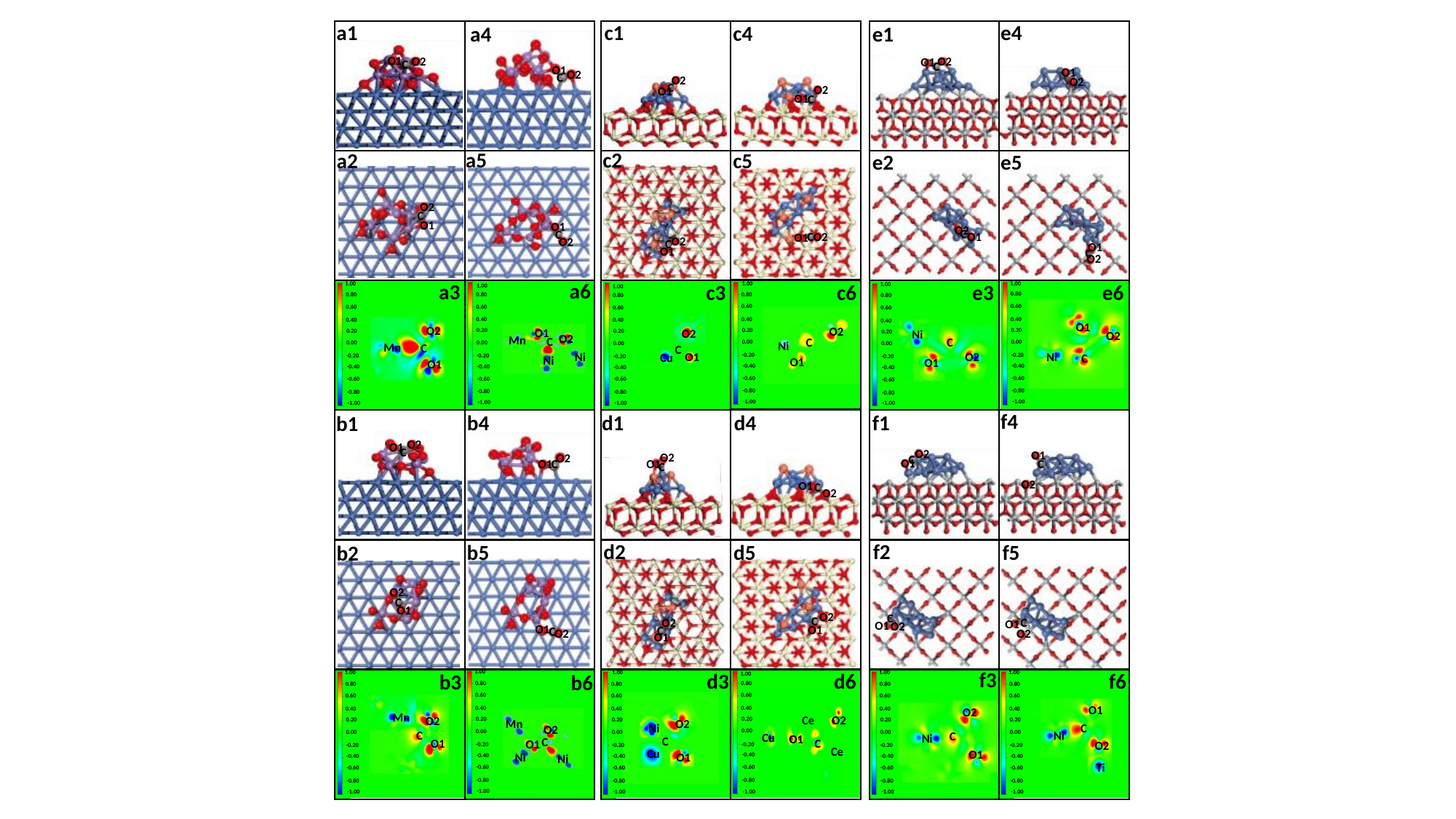

e4
a1
c1
c4
e1
a4
c2
a5
a2
c5
e2
e5
a6
a3
c6
e6
c3
e3
f4
b4
f1
d4
d1
b1
f2
d2
f5
b5
d5
b2
f3
d6
d3
f6
b3
b6
O1
O2
O2
O1
C
C
O1
O1
O2
C
O2
O2
C
O2
O1
O1
C
O2
C
O1
O1
O2
C
C
O2
O1
C
O1
O2
O2
C
O1
O1
C
O2
1.00
1.00
1.00
1.00
1.00
1.00
0.80
0.80
0.80
0.80
0.80
0.80
0.60
0.60
0.60
0.60
0.60
0.60
0.40
0.40
0.40
0.40
0.40
0.40
O1
O2
O2
O1
0.20
0.20
0.20
O2
0.20
0.20
0.20
Ni
O2
O2
Mn
C
C
C
0.00
0.00
Ni
0.00
0.00
0.00
0.00
Mn
C
C
Ni
O1
Ni
O2
Cu
-0.20
-0.20
C
-0.20
-0.20
-0.20
-0.20
Ni
O1
O1
O1
-0.40
-0.40
-0.40
-0.40
-0.40
-0.40
-0.60
-0.60
-0.60
-0.60
-0.60
-0.60
-0.80
-0.80
-0.80
-0.80
-0.80
-0.80
-1.00
-1.00
-1.00
-1.00
-1.00
-1.00
O2
O1
C
O2
O1
O2
O2
C
O1
O1
C
C
O1
C
O2
O1
C
O2
O2
C
O1
O2
C
C
C
O2
O1
O1
O2
O1
O1
C
C
O2
O2
O1
1.00
1.00
1.00
1.00
1.00
1.00
0.80
0.80
0.80
0.80
0.80
0.80
0.60
0.60
0.60
0.60
0.60
0.60
O1
0.40
0.40
0.40
0.40
0.40
O2
0.40
Mn
Ce
O2
O2
0.20
0.20
0.20
0.20
0.20
0.20
Mn
O2
C
Ni
O2
0.00
0.00
0.00
0.00
0.00
0.00
C
Ni
C
Cu
Ni
O1
C
C
O1
C
O1
O2
-0.20
-0.20
-0.20
-0.20
-0.20
-0.20
Ce
Cu
O1
O1
Ni
-0.40
-0.40
Ni
-0.40
-0.40
-0.40
-0.40
Ti
-0.60
-0.60
-0.60
-0.60
-0.60
-0.60
-0.80
-0.80
-0.80
-0.80
-0.80
-0.80
-1.00
-1.00
-1.00
-1.00
-1.00
-1.00
