# Supplementary material for: Enhanced carbon dioxide electrolysis at redox manipulated interfaces
Source: Nat Commun. 2019 Apr 4;10:1550. doi: 10.1038/s41467-019-09568-1 (PMC6449360; doi:10.1038/s41467-019-09568-1)
Supplement: Supplementary file 3 — Source Data [file 41467_2019_9568_MOESM3_ESM.zip › Source Data-20190315/Supplementary Table 1/Supplementary Table 1.docx]

**Supplementary Table 1** Vibrational frequencies in cm^-1^ of CO_2_ species and CO_3_^2-^ species adsorbed on MnO, CeO_2_ and TiO_2_ surfaces.

| system | species | parameter | Figure | C-O (Å) | υ (cm^-1^) |
| --- | --- | --- | --- | --- | --- |
| MnO | CO_2_ | experimental | **-** | **-** | 2361 |
|  |  | computational | 7a | 1.17 | 2372 |
|  | CO_3_^2-^ | experimental | - | **-** | 1382 |
|  |  | computational | 7b | 1.31 | 1364 |
| CeO_2_ | CO_2_ | experimental | - | **-** | 2360 |
|  |  | computational | 7c | 1.17 | 2372 |
|  | CO_3_^2-^ | experimental | - | **-** | 1389 |
|  |  | computational | 7d | 1.26 | 1390 |
| TiO_2_ | CO_2_ | experimental | - | **-** | 2359 |
|  |  | computational | 7e | 1.18 | 2355 |
|  | CO_3_^2-^ | experimental | - | **-** | 1397 |
|  |  | computational | 7f | 1.29 | 1398 |
