# Supplementary material for: Enhanced carbon dioxide electrolysis at redox manipulated interfaces
Source: Nat Commun. 2019 Apr 4;10:1550. doi: 10.1038/s41467-019-09568-1 (PMC6449360; doi:10.1038/s41467-019-09568-1)
Supplement: Supplementary file 3 — Source Data [file 41467_2019_9568_MOESM3_ESM.zip › Source Data-20190315/Supplementary Table 2/Supplementary Table 2.docx]

**Supplementary Table 2** Geometrical parameters and calculated adsorption energies of CO_2_ species on MnO_x_/Ni(111) surface. Mn_6_O_12_/Ni-I and Mn_6_O_12_/Ni-II are the adsorption configurations of Figure 5 a1 and a4, Mn_6_O_9_/Ni-I and Mn_6_O_9_/Ni-II are the adsorption configurations of Figure 5 b1 and b4.

| parameter | CO_2_ | Mn_6_O_12_/Ni-I | Mn_6_O_12_/Ni-II | Mn_6_O_10_/Ni | Mn_6_O_9_/Ni-I | Mn_6_O_9_/Ni-II |
| --- | --- | --- | --- | --- | --- | --- |
| C-Ni (Å) | - | - | 1.87 | 1.91 | 1.89 | - |
| C-Mn (Å) | - | 2.03 | - | - | - | 2.19 |
| Mn-O1 (Å) | - | 1.86 | 2.05 | 1.96 | 1.94 | - |
| Ni-O1 (Å) | - | - | - | 2.00 | 2.00 | - |
| Ni-O2 (Å) | - | - | 2.03 | - | - | - |
| C-O1 (Å) | 1.18 | 1.33 | 1.27 | 1.38 | 1.39 | 1.38 |
| C-O2 (Å) | 1.18 | 1.21 | 1.28 | 1.21 | 1.21 | 1.21 |
| O-C-O (°) | 180 | 130.4 | 127.2 | 124.4 | 124.0 | 124.0 |
| E_ads_ (eV) | - | -0.98 | -1.97 | -2.10 | -2.64 | -1.62 |
