# Supplementary material for: Enhanced carbon dioxide electrolysis at redox manipulated interfaces
Source: Nat Commun. 2019 Apr 4;10:1550. doi: 10.1038/s41467-019-09568-1 (PMC6449360; doi:10.1038/s41467-019-09568-1)
Supplement: Supplementary file 3 — Source Data [file 41467_2019_9568_MOESM3_ESM.zip › Source Data-20190315/Supplementary Table 3/Supplementary Table 3.docx]

**Supplementary Table 3** Geometrical parameters and calculated adsorption energies of CO_2_ species on the M/CeO_2_(111) systems. (M is expressed as Cu, Ni or Ni-Cu clusters).

| parameter | CO_2_ | Cu/CeO_2_ | Ni/CeO_2_ | Ni-Cu/CeO_2_ | Cu/CeO_2-x_ | Ni/CeO_2-x_ | (Ni-Cu)/CeO_2-x_ |
| --- | --- | --- | --- | --- | --- | --- | --- |
| C-O (Å) | - | 1.36 | 1.36 | 1.36 | - | - | - |
| C-Ni (Å) | - | - | - | - | - | 1.85 | 1.85 |
| C-Cu (Å) | - | 1.93 | - | - | 1.94 | - | - |
| Ni-O1 (Å) | - | - | - | - | - | - | 1.86 |
| Ni-O2 (Å) | - | - | 1.86 | - | - | - | - |
| Cu-O1 (Å) | - | 1.84 | - | 1.84 | - | - | - |
| Ce-O1 (Å) | - | - | - | - | - | - | 2.57 |
| Ce-O2 (Å) | - | 2.41 | 2.43 | - | 2.50/2.55 | 2.58/2.60 | 2.59/2.62 |
| C-O1 (Å) | 1.18 | 1.28 | 1.27 | 1.27 | 1.24 | 1.23 | 1.23 |
| C-O2 (Å) | 1.18 | 1.27 | 1.28 | 1.21 | 1.36 | 1.35 | 1.35 |
| O-C-O (°) | 180 | 123.3 | 123.2 | 124.0 | 117.5 | 116.3 | 123.2 |
| E_ads_ (eV) | - | -1.32 | -1.73 | -2.06 | -1.83 | -1.90 | -2.18 |
