# Supplementary material for: Enhanced carbon dioxide electrolysis at redox manipulated interfaces
Source: Nat Commun. 2019 Apr 4;10:1550. doi: 10.1038/s41467-019-09568-1 (PMC6449360; doi:10.1038/s41467-019-09568-1)
Supplement: Supplementary file 3 — Source Data [file 41467_2019_9568_MOESM3_ESM.zip › Source Data-20190315/Supplementary Table 4/Supplementary Table 4.docx]

**Supplementary** **Table 4** Geometrical parameters and calculated adsorption energies of CO_2_ species on Ni/TiO_2_(101) systems. TiO_2_-I to TiO_2_-IV are the adsorption configurations of Figure 9 e1 to e4, TiO_2-x_-I and TiO_2-x_-II are the adsorption configurations of Figure 5 f1 and f4.

| parameter | CO_2_ | TiO_2_-I | TiO_2_-II | TiO_2_-III | TiO_2_-IV | TiO_2-x_-I | TiO_2-x_-II |
| --- | --- | --- | --- | --- | --- | --- | --- |
| C-Ni (Å) | - | 1.93 | 2.06 | 1.96 | - | 1.87 | 1.78/1.87 |
| O1-Ni (Å) | - | 1.97 | 1.97 | 1.95 | - | 2.01 | - |
| O2-Ni (Å) | - | 1.97 | - | 1.95 | - | 1.99 | - |
| O-Ti (Å) | - | - | 2.15 | - | 2.04/1.99 | - | 1.67 |
| C-O1 (Å) | 1.18 | 1.28 | 1.28 | 1.29 | 1.20 | 1.26 | 1.22 |
| C-O2 (Å) | 1.18 | 1.27 | 1.28 | 1.30 | 1.28 | 1.29 | 3.85 |
| O-C-O (°) | 180 | 130.8 | 126.0 | 127.2 | 134.3 | 131.2 | 106.8 |
| E_ads_ (eV) | - | -1.39 | -1.27 | -1.44 | -0.25 | -1.16 | -1.94 |
