# Supplementary material for: Enhanced carbon dioxide electrolysis at redox manipulated interfaces
Source: Nat Commun. 2019 Apr 4;10:1550. doi: 10.1038/s41467-019-09568-1 (PMC6449360; doi:10.1038/s41467-019-09568-1)
Supplement: Supplementary file 3 — Source Data [file 41467_2019_9568_MOESM3_ESM.zip › Source Data-20190315/Supplementary Table 5/Supplementary Table 5.docx]

**Supplementary Table 5** The energy values and adsorption energy of CO_2_ with different cut-off energy. TiO_2_-I to TiO_2_-IV are the adsorption configurations of Figure 9 e1 to e4.

| Cut-off (eV) | 300 | 350 | 400 | 450 | 500 | 550 | 600 | 650 |
| --- | --- | --- | --- | --- | --- | --- | --- | --- |
| $\mathbf{E}_{\mathbf{C}\mathbf{O}_{\mathbf{2}}}$ (eV) | -23.16 | -23.02 | -23.98 | -22.95 | -22.94 | -22.95 | -22.96 | -22.97 |
| $\mathbf{E}_{\mathbf{T}\mathbf{iO}_{\mathbf{2}}}$ (eV) | -1309.35 | -1300.10 | -1296.86 | -1296.48 | -1296.18 | -1296.19 | -1296.47 | -1296.77 |
| $\mathbf{E}_{\mathbf{T}\mathbf{iO}_{\mathbf{2}}}$-I (eV) | -1333.91 | -1324.54 | -1322.24 | -1320.82 | -1320.45 | -1320.53 | -1320.82 | -1321.13 |
| $\mathbf{E}_{\mathbf{T}\mathbf{iO}_{\mathbf{2}}}$-II (eV) | -1333.79 | -1324.02 | -1322.12 | -1320.70 | -1320.19 | -1320.40 | -1320.63 | -1321.01 |
| $\mathbf{E}_{\mathbf{T}\mathbf{iO}_{\mathbf{2}}}$-III (eV) | -1333.97 | -1324.60 | -1322.28 | -1320.87 | -1320.50 | -1320.58 | -1320.87 | -1321.15 |
| $\mathbf{E}_{\mathbf{T}\mathbf{iO}_{\mathbf{2}}}$-IV (eV) | -1332.82 | -1323.41 | -1321.09 | -1319.68 | -1319.30 | -1319.38 | -1319.68 | -1319.98 |
| $\mathbf{E}_{\mathbf{ads}}$-I (eV) | -1.41 | -1.42 | -1.40 | -1.39 | -1.33 | -1.39 | -1.39 | -1.39 |
| $\mathbf{E}_{\mathbf{ads}}$-II (eV) | -1.29 | -0.90 | -1.29 | -1.27 | -1.07 | -1.26 | -1.20 | -1.27 |
| $\mathbf{E}_{\mathbf{ads}}$-III (eV) | -1.47 | -1.47 | -1.45 | -1.44 | -1.37 | -1.43 | -1.44 | -1.41 |
| $\mathbf{E}_{\mathbf{ads}}$-IV (eV) | -0.32 | -0.29 | -0.26 | -0.25 | -0.18 | -0.24 | -0.25 | -0.24 |
