# Supplementary figures and images for: Enhanced carbon dioxide electrolysis at redox manipulated interfaces
Source: Nat Commun. 2019 Apr 4;10:1550. doi: 10.1038/s41467-019-09568-1 (PMC6449360; doi:10.1038/s41467-019-09568-1)

## Slide 1
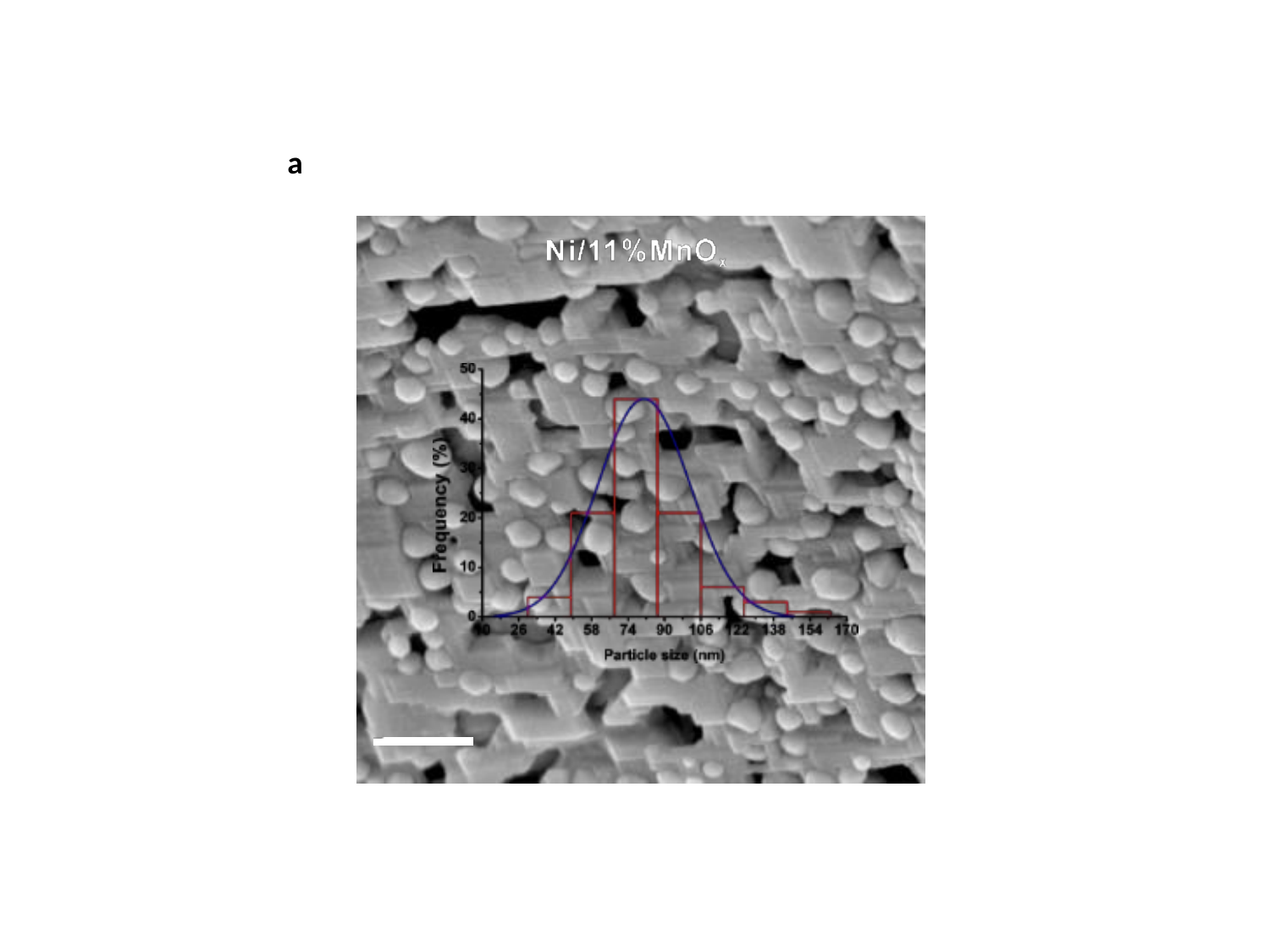

a

## Slide 2
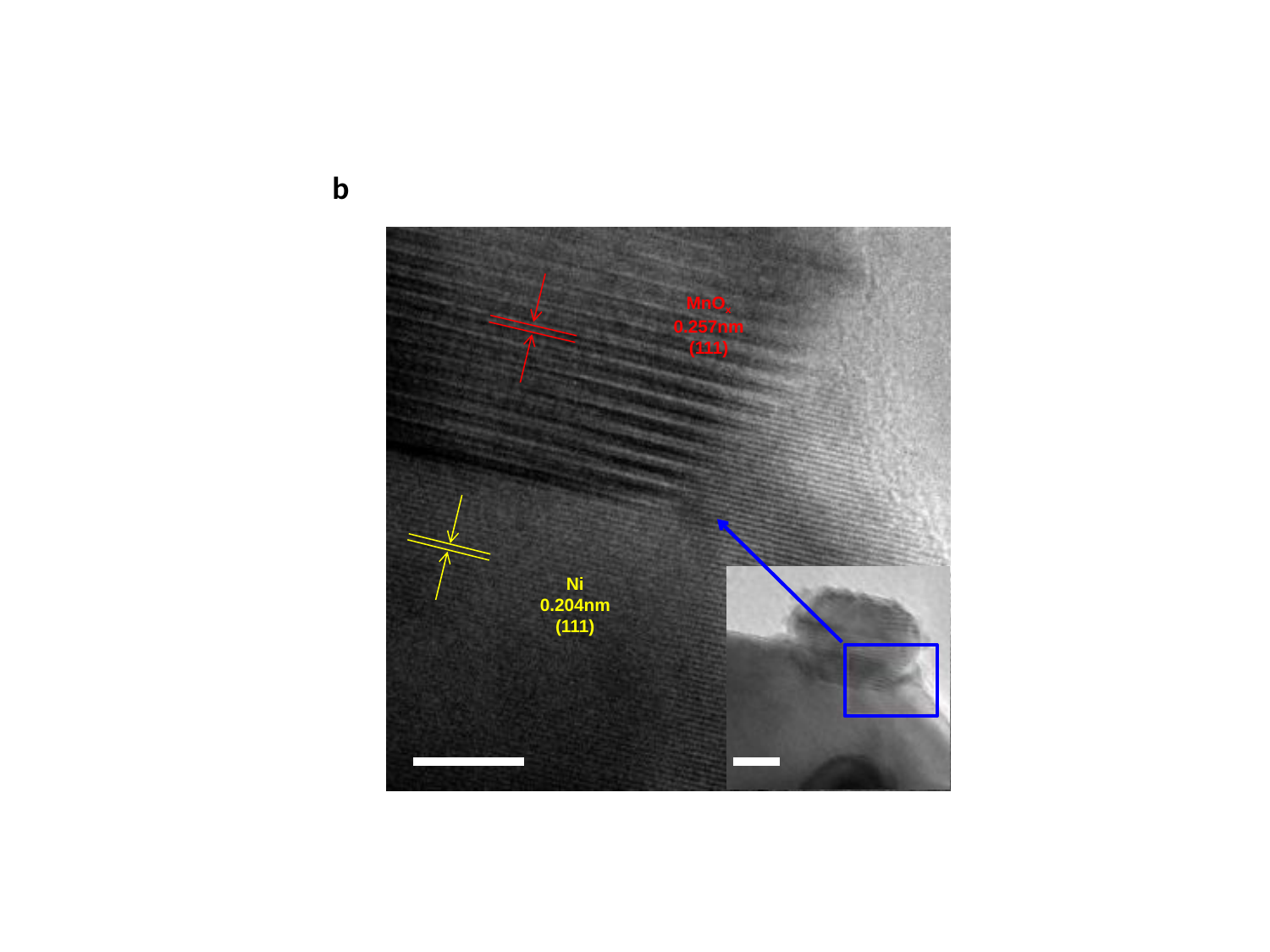

b
MnOx
0.257nm
(111)
Ni
0.204nm
(111)

## Slide 3
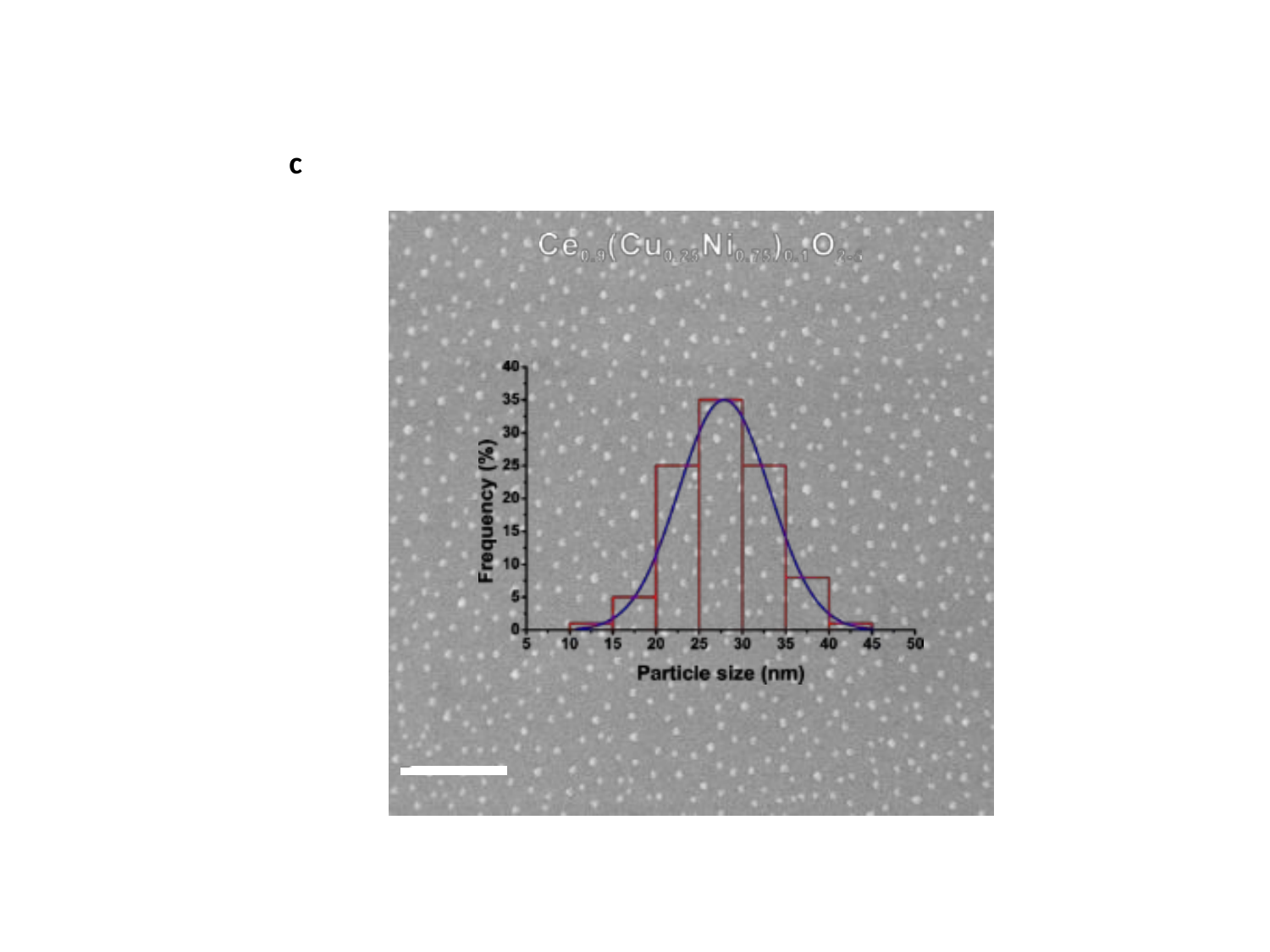

c

## Slide 4
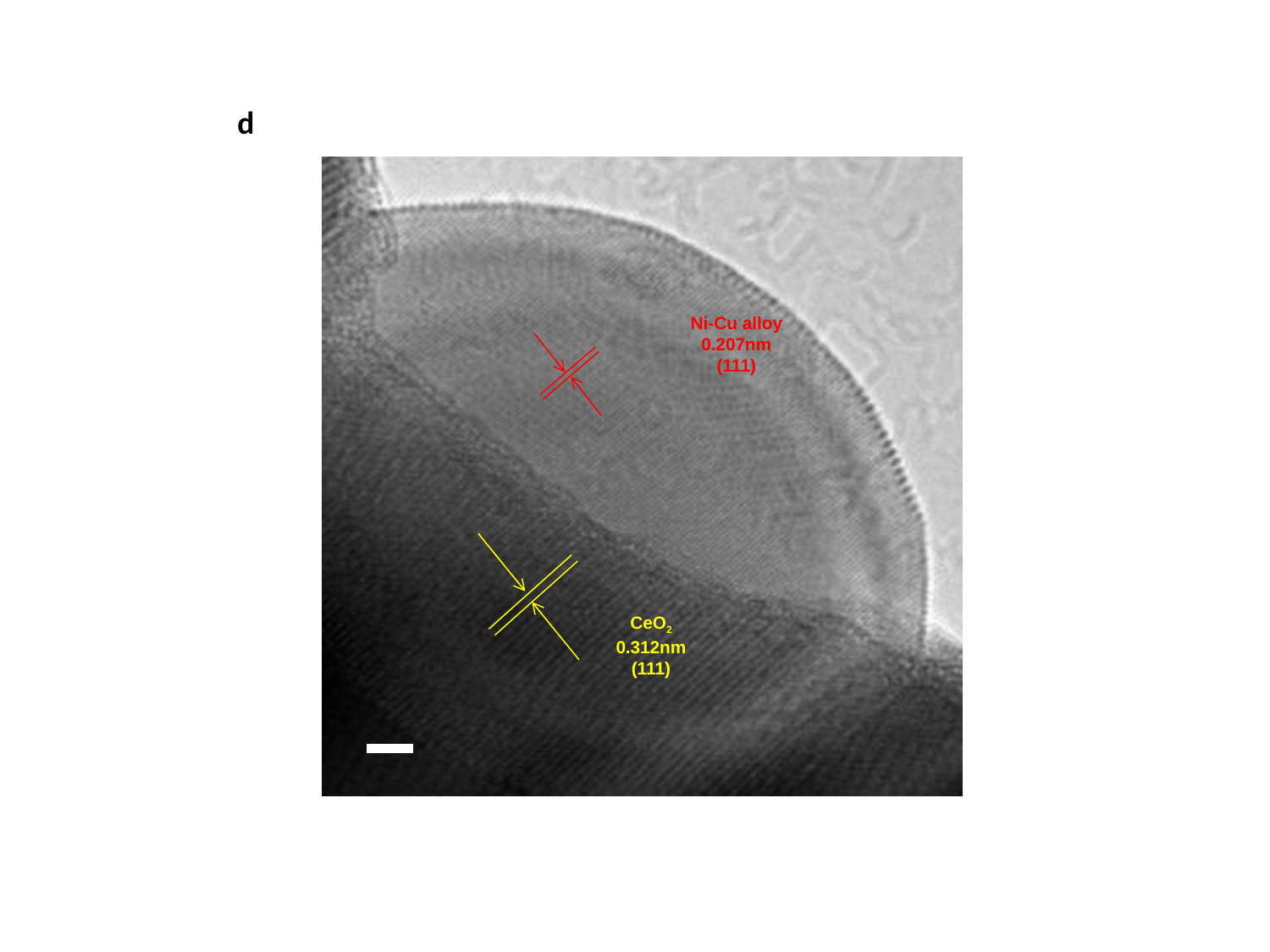

d
Ni-Cu alloy
0.207nm
(111)
CeO2
0.312nm
(111)

## Slide 5
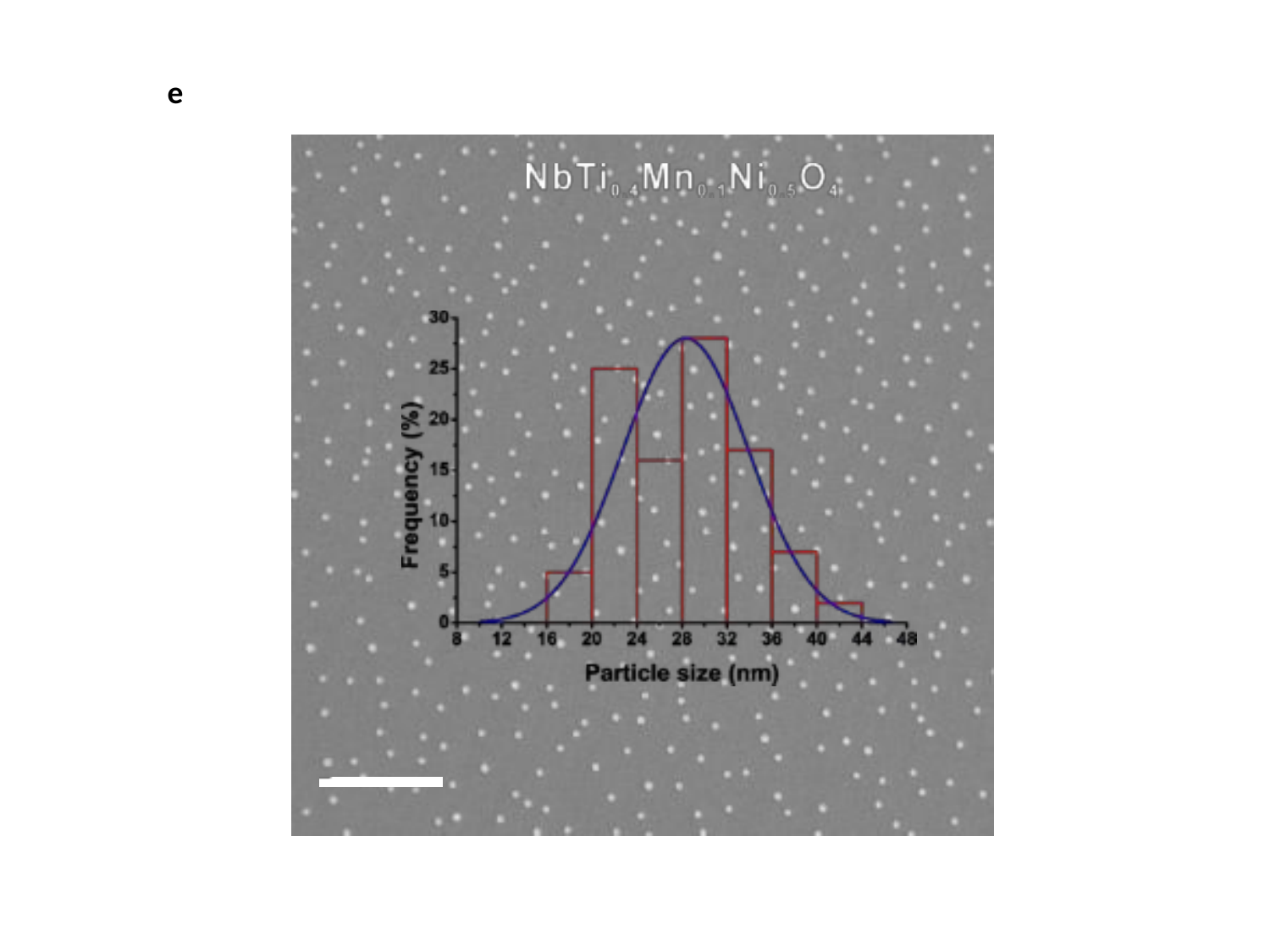

e

## Slide 6
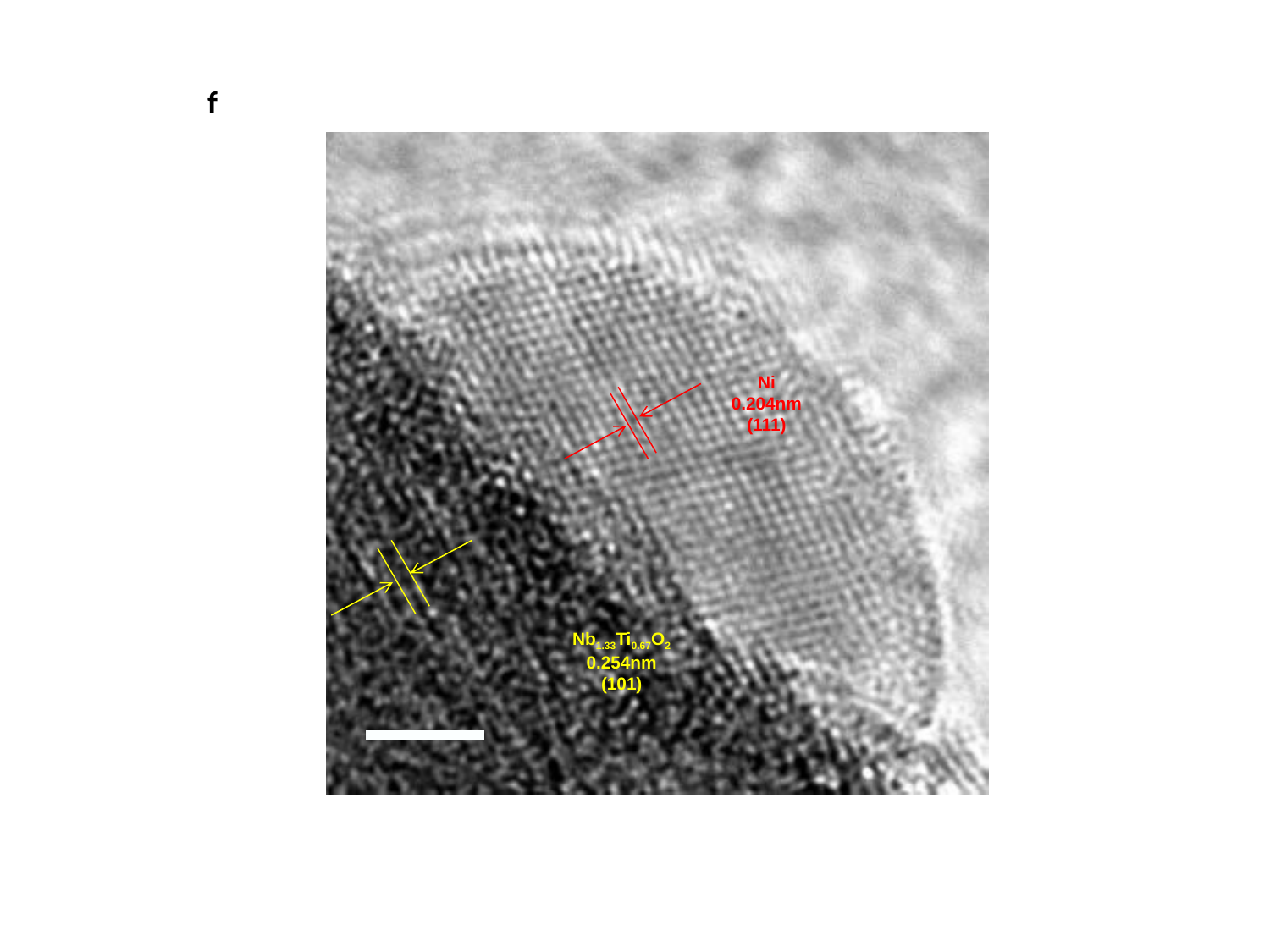

f
Ni
0.204nm
(111)
Nb1.33Ti0.67O2
0.254nm
(101)

Supplement: Supplementary file 3 — Source Data [file 41467_2019_9568_MOESM3_ESM.zip › Source Data-20190315/Figure 2/Figure 2.pptx]

## Slide 1
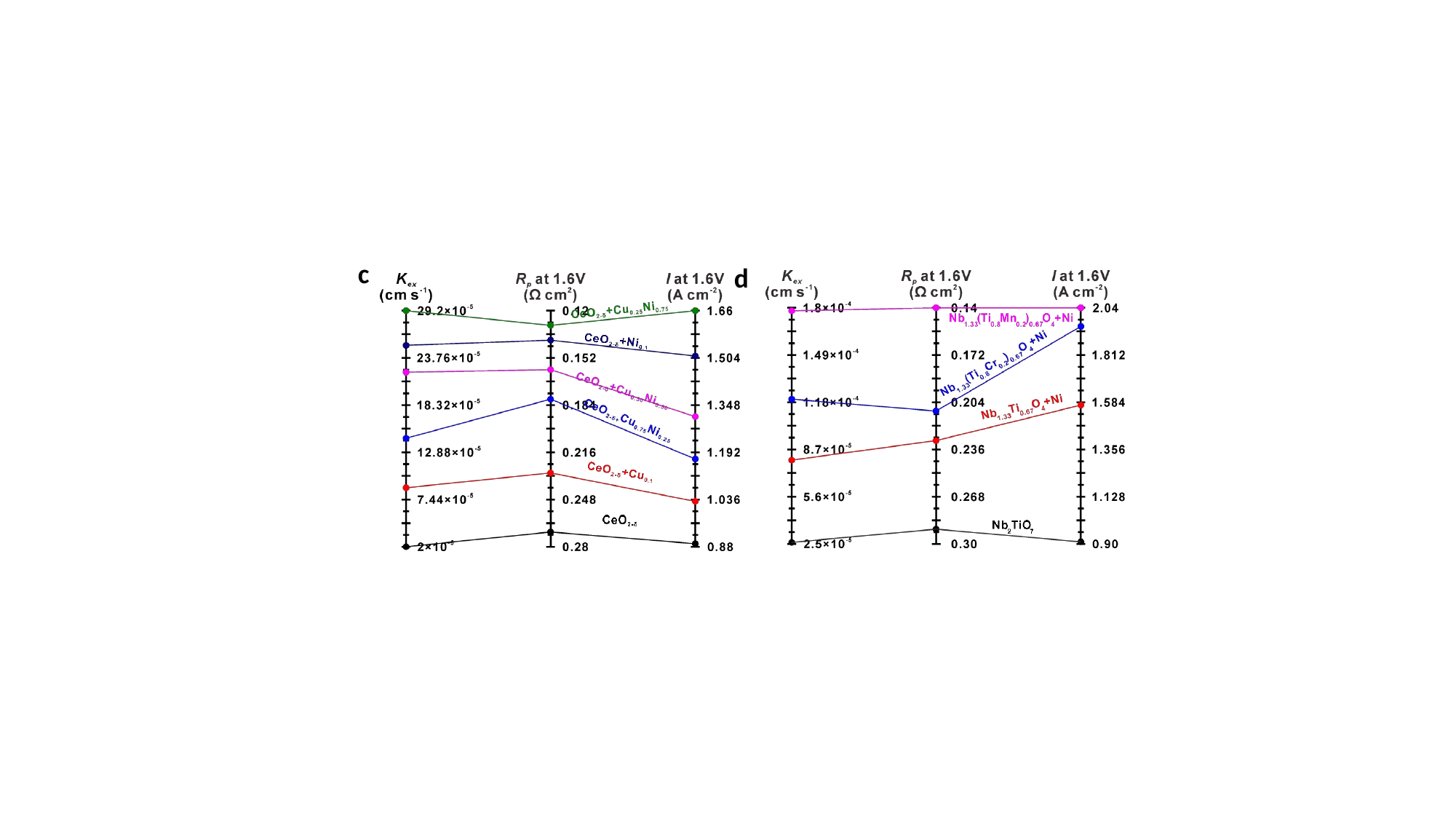

c
d

Supplement: Supplementary file 3 — Source Data [file 41467_2019_9568_MOESM3_ESM.zip › Source Data-20190315/Figure 3/Figure 3c-d.pptx]

## Slide 1
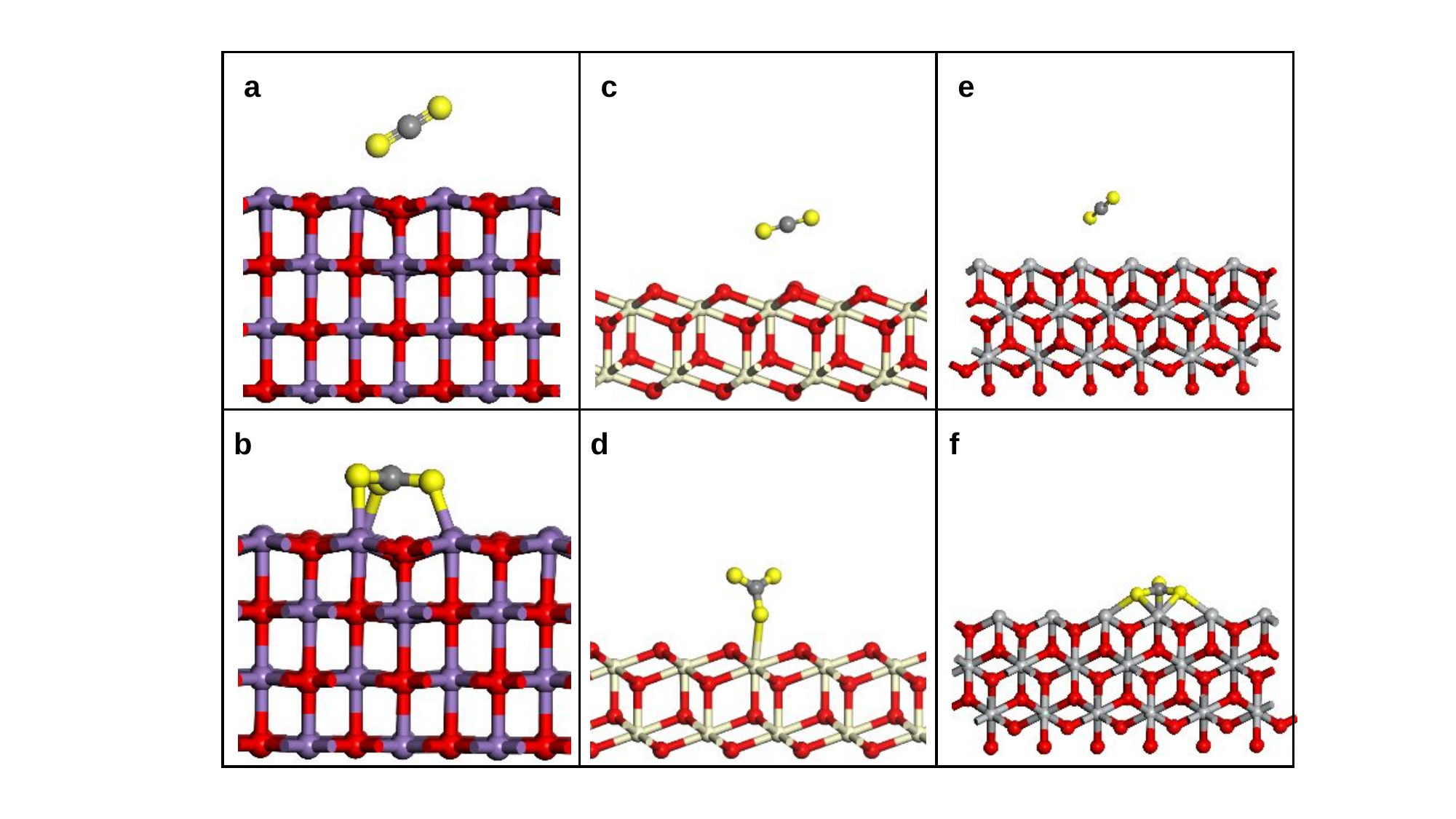

a
c
e
b
d
f

Supplement: Supplementary file 3 — Source Data [file 41467_2019_9568_MOESM3_ESM.zip › Source Data-20190315/Supplementary Figure 7/Supplementary Figure 7.pptx]

## Slide 1
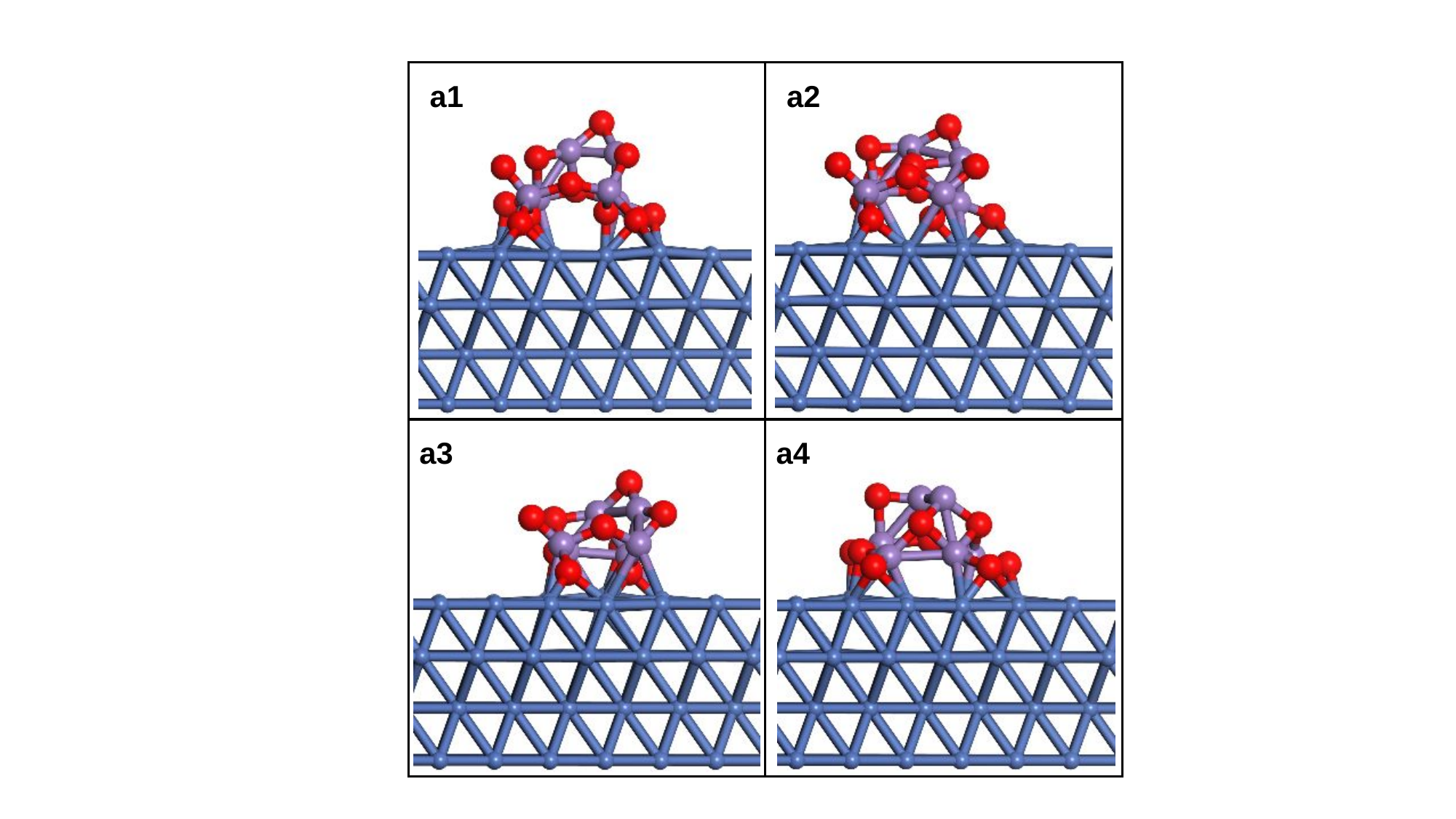

a1
a2
a3
a4

Supplement: Supplementary file 3 — Source Data [file 41467_2019_9568_MOESM3_ESM.zip › Source Data-20190315/Supplementary Figure 8/Supplementary Figure 8a.pptx]

## Slide 1
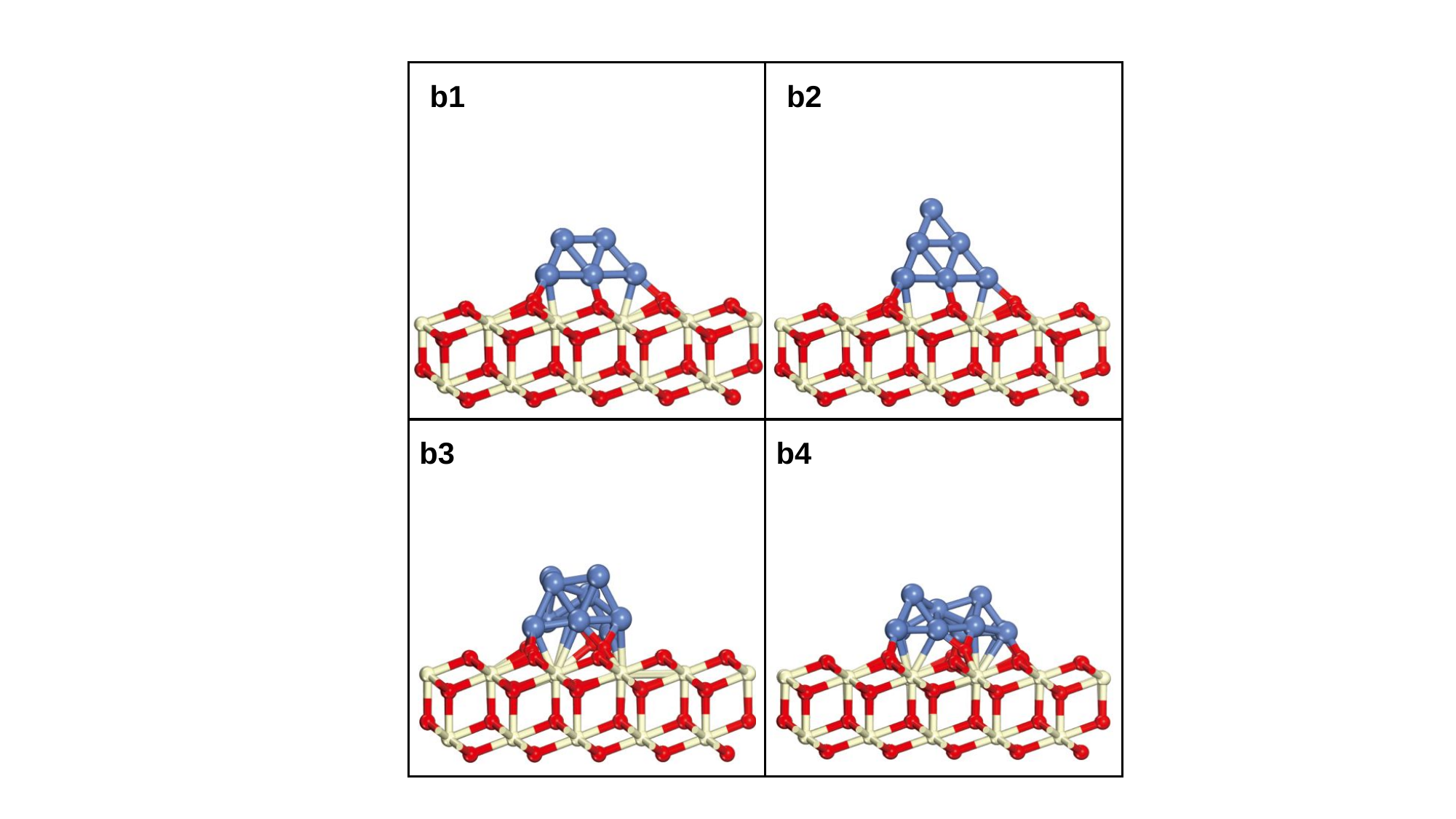

b1
b2
b3
b4

Supplement: Supplementary file 3 — Source Data [file 41467_2019_9568_MOESM3_ESM.zip › Source Data-20190315/Supplementary Figure 8/Supplementary Figure 8b.pptx]

## Slide 1
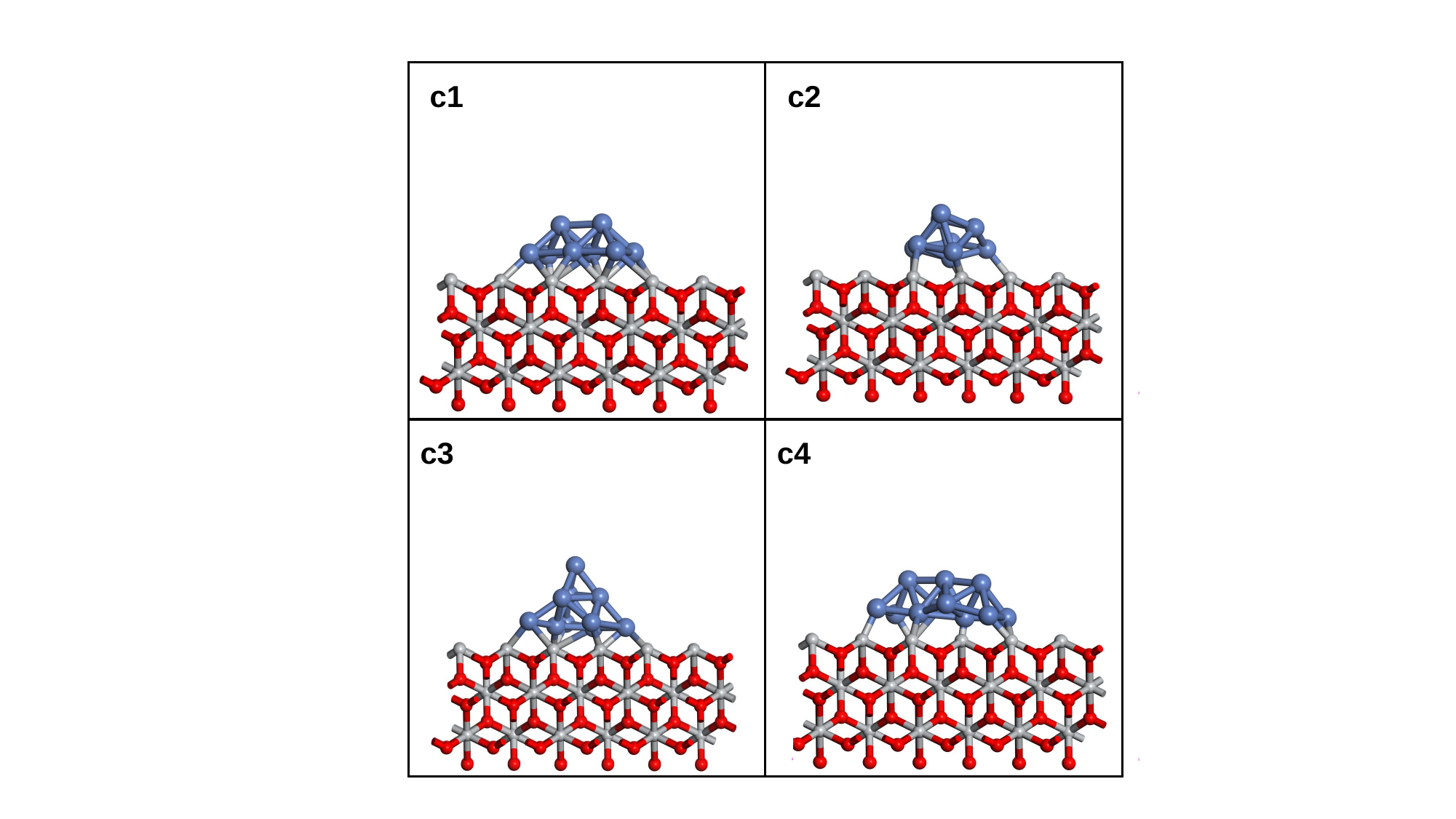

c1
c2
c3
c4

Supplement: Supplementary file 3 — Source Data [file 41467_2019_9568_MOESM3_ESM.zip › Source Data-20190315/Supplementary Figure 8/Supplementary Figure 8c.pptx]

## Slide 1
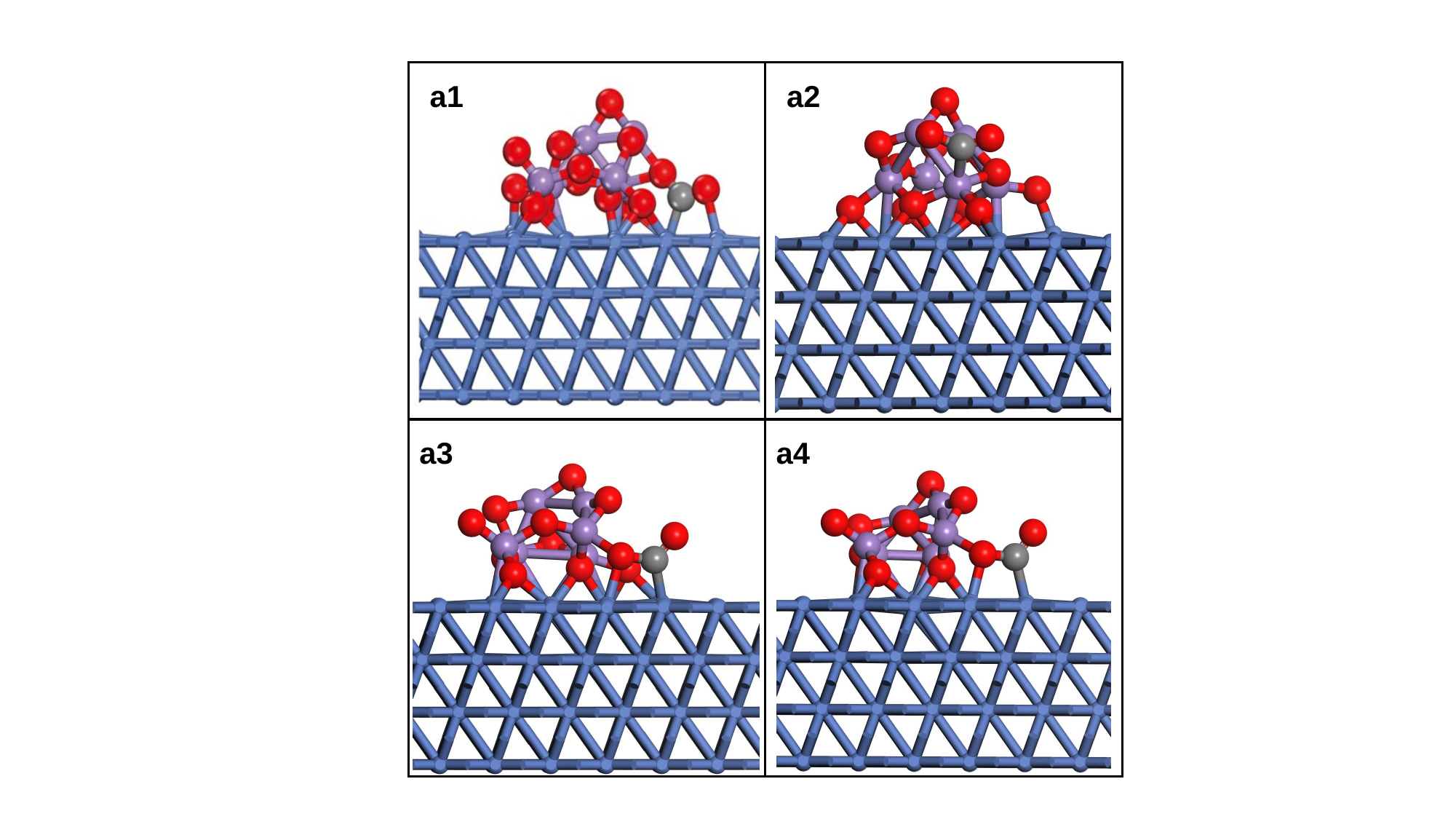

a1
a2
a3
a4

Supplement: Supplementary file 3 — Source Data [file 41467_2019_9568_MOESM3_ESM.zip › Source Data-20190315/Supplementary Figure 9/Supplementary Figure 9a.pptx]

## Slide 1
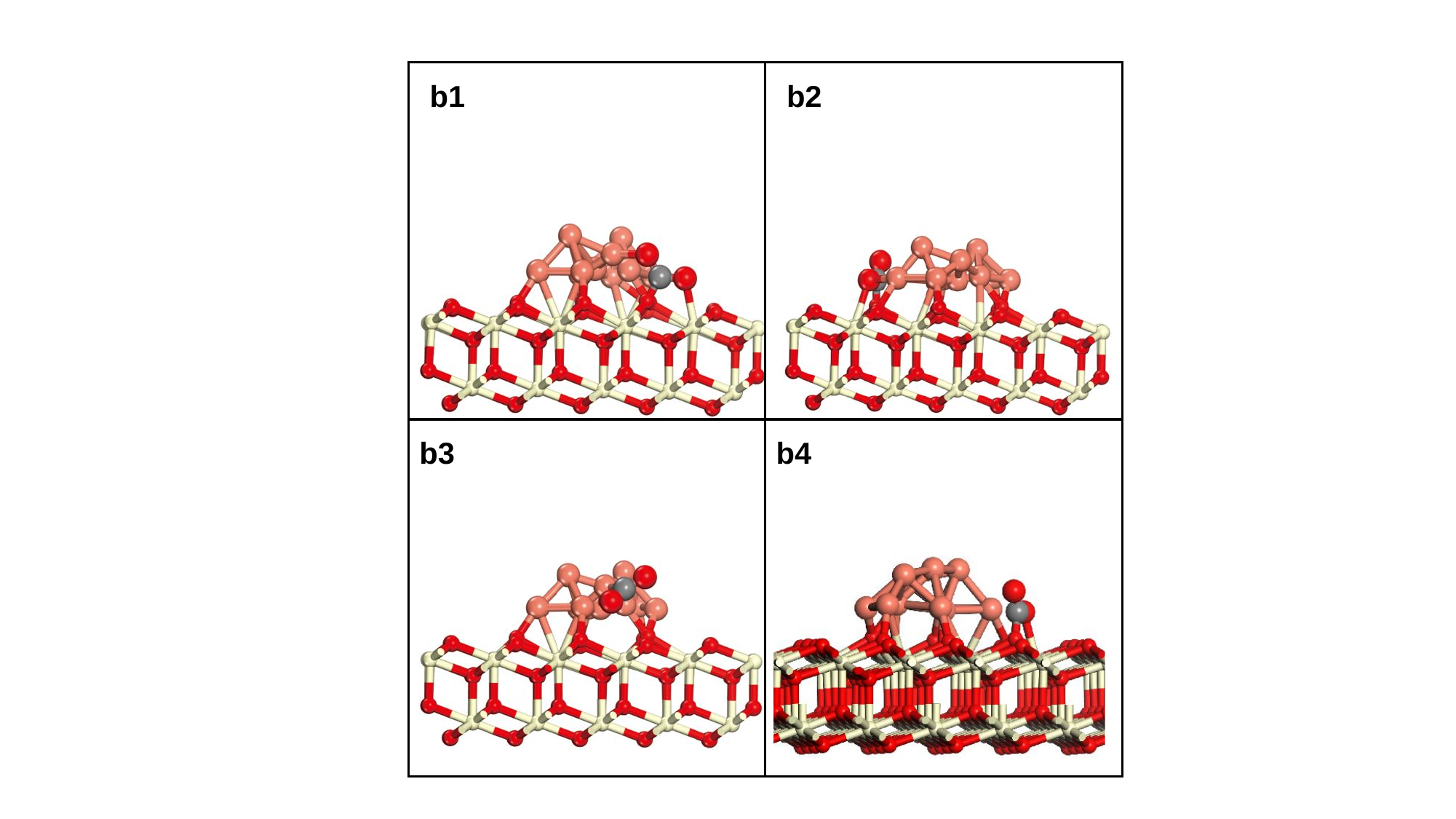

b1
b2
b3
b4

Supplement: Supplementary file 3 — Source Data [file 41467_2019_9568_MOESM3_ESM.zip › Source Data-20190315/Supplementary Figure 9/Supplementary Figure 9b.pptx]

## Slide 1
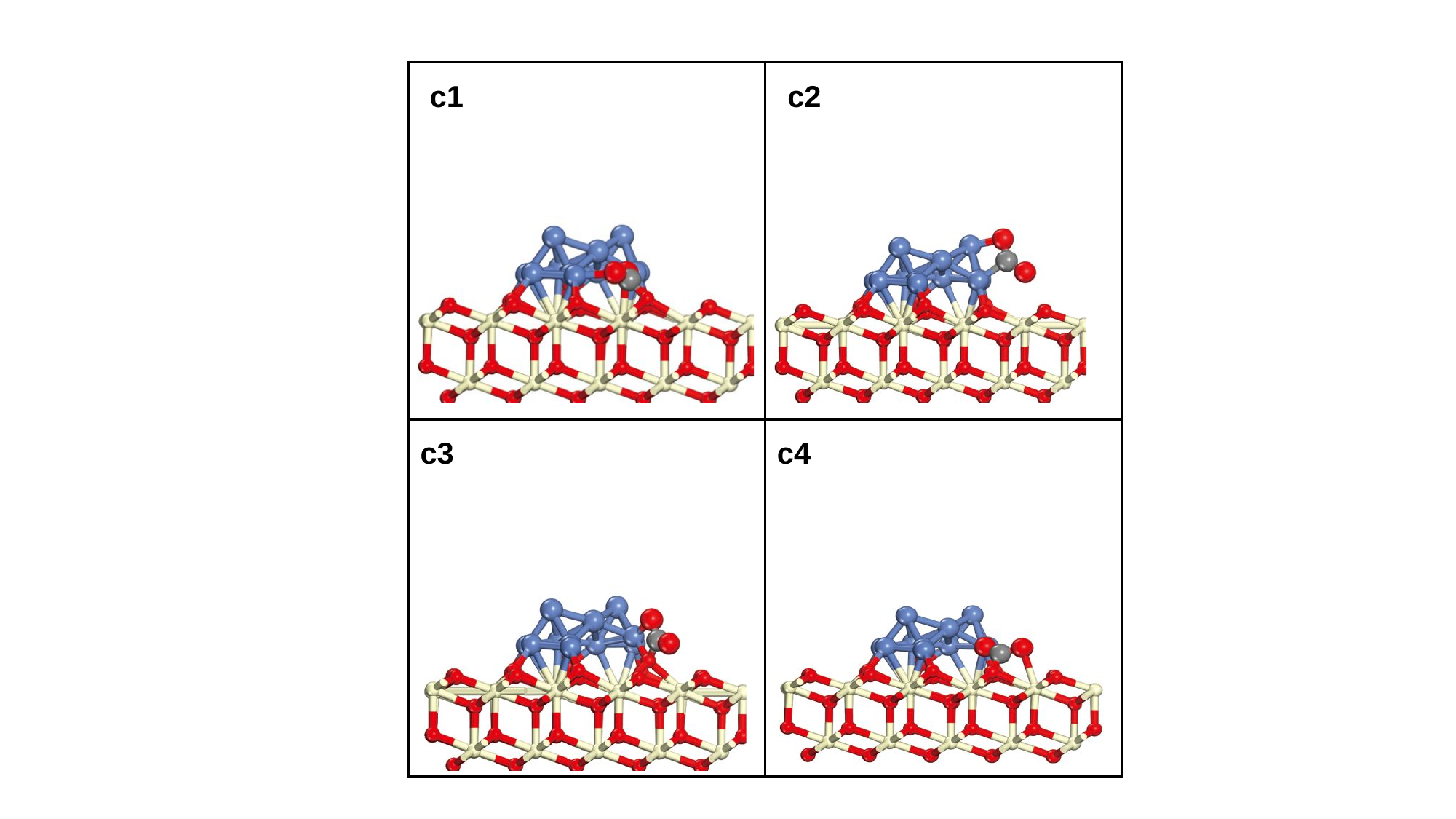

c1
c2
c3
c4

Supplement: Supplementary file 3 — Source Data [file 41467_2019_9568_MOESM3_ESM.zip › Source Data-20190315/Supplementary Figure 9/Supplementary Figure 9c.pptx]

## Slide 1
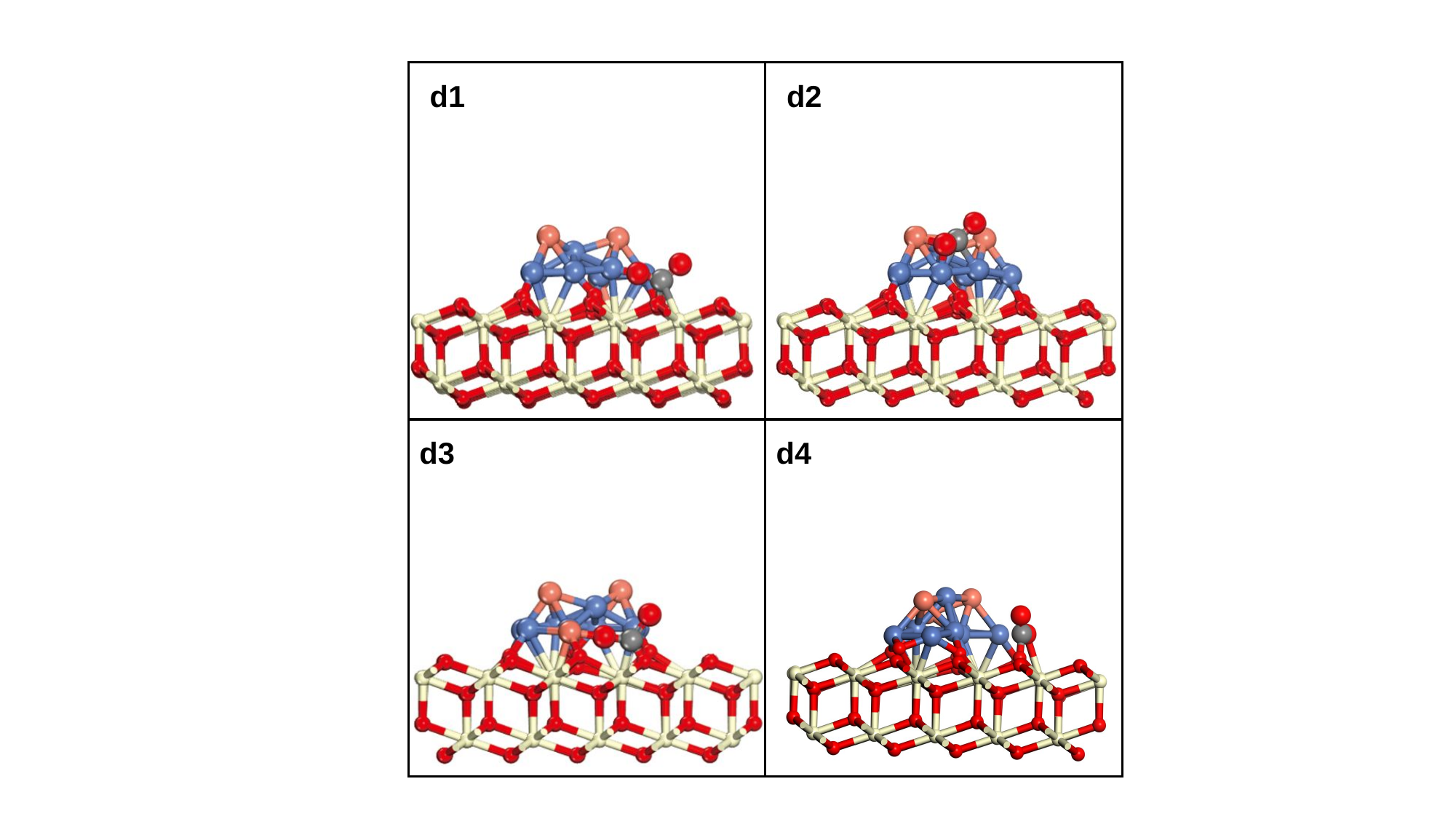

d1
d2
d3
d4

Supplement: Supplementary file 3 — Source Data [file 41467_2019_9568_MOESM3_ESM.zip › Source Data-20190315/Supplementary Figure 9/Supplementary Figure 9d.pptx]

## Slide 1
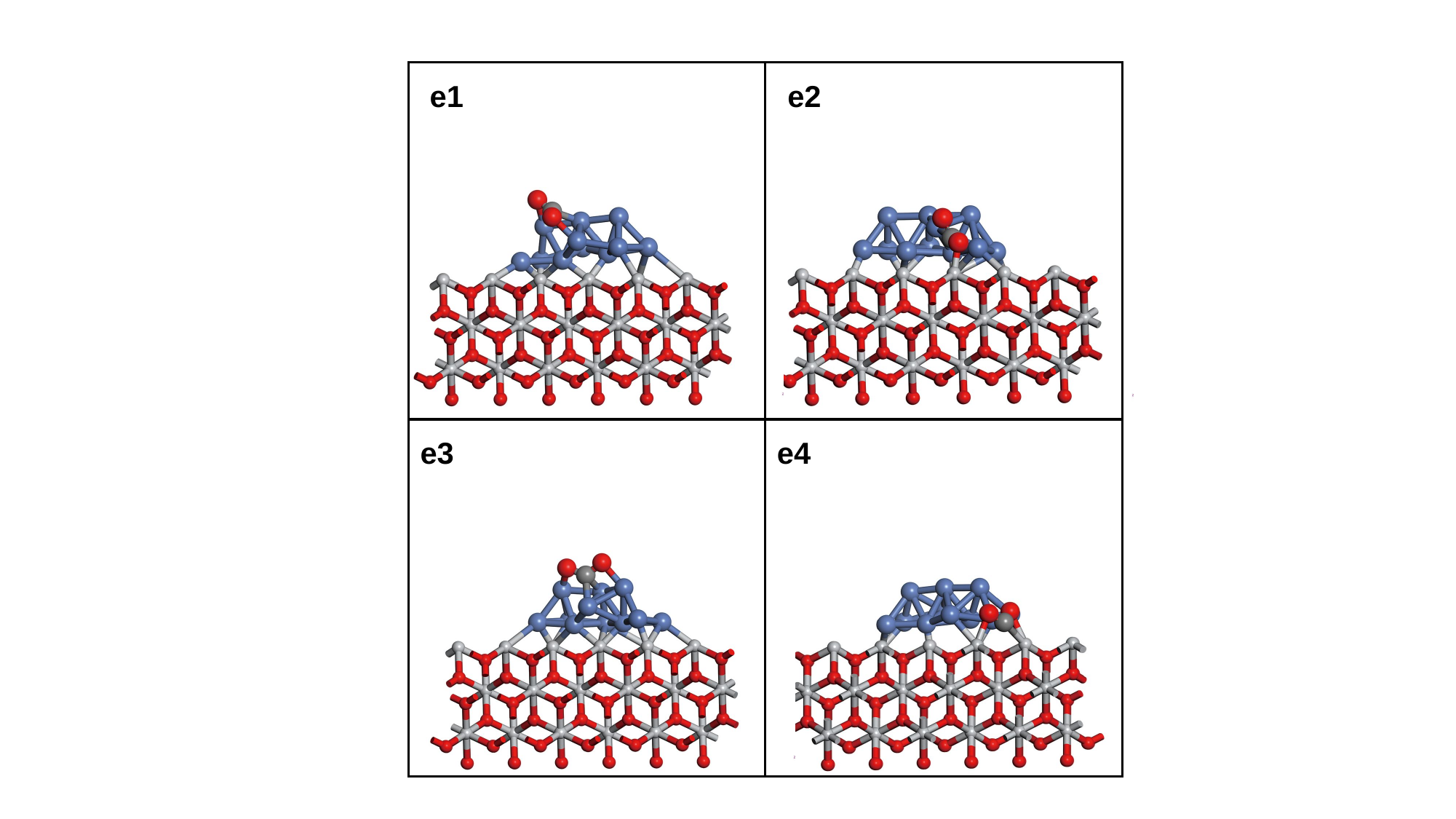

e1
e2
e3
e4

Supplement: Supplementary file 3 — Source Data [file 41467_2019_9568_MOESM3_ESM.zip › Source Data-20190315/Supplementary Figure 9/Supplementary Figure 9e.pptx]
